# Sedentary behaviour and risk of all-cause, cardiovascular and cancer mortality, and incident type 2 diabetes: a systematic review and dose response meta-analysis

**DOI:** 10.1007/s10654-018-0380-1

**Published:** 2018-03-28

**Authors:** Richard Patterson, Eoin McNamara, Marko Tainio, Thiago Hérick de Sá, Andrea D. Smith, Stephen J. Sharp, Phil Edwards, James Woodcock, Søren Brage, Katrien Wijndaele

**Affiliations:** 1 Public Health Policy Evaluation Unit, School of Public Health, Imperial College London, London, W6 8RP UK; 2 MRC Epidemiology Unit, School of Clinical Medicine, University of Cambridge, Cambridge, CB2 0QQ UK; 3 Centre for Epidemiological Research in Nutrition and Health, University of São Paulo, São Paulo, Brazil; 4 Research Department of Behavioural Science and Health, University College London, London, WC1E 6BT UK; 5 Faculty of Epidemiology and Population Health, London School of Hygiene and Tropical Medicine, London, WC1E 7HT UK

**Keywords:** Sedentary, Prevention, Meta-analysis, Public health, Mortality, Diabetes

## Abstract

*Purpose:* To estimate the strength and shape of the dose–response relationship between sedentary behaviour and all-cause, cardiovascular disease (CVD) and cancer mortality, and incident type 2 diabetes (T2D), adjusted for physical activity (PA). *Data Sources:* Pubmed, Web of Knowledge, Medline, Embase, Cochrane Library and Google Scholar (through September-2016); reference lists. *Study Selection:* Prospective studies reporting associations between total daily sedentary time or TV viewing time, and ≥ one outcome of interest. *Data Extraction:* Two independent reviewers extracted data, study quality was assessed; corresponding authors were approached where needed. *Data Synthesis:* Thirty-four studies (1,331,468 unique participants; good study quality) covering 8 exposure-outcome combinations were included. For total sedentary behaviour, the PA-adjusted relationship was non-linear for all-cause mortality (RR per 1 h/day: were 1.01 (1.00–1.01) ≤ 8 h/day; 1.04 (1.03–1.05) > 8 h/day of exposure), and for CVD mortality (1.01 (0.99–1.02) ≤ 6 h/day; 1.04 (1.03–1.04) > 6 h/day). The association was linear (1.01 (1.00–1.01)) with T2D and non-significant with cancer mortality. Stronger PA-adjusted associations were found for TV viewing (h/day); non-linear for all-cause mortality (1.03 (1.01–1.04) ≤ 3.5 h/day; 1.06 (1.05–1.08) > 3.5 h/day) and for CVD mortality (1.02 (0.99–1.04) ≤ 4 h/day; 1.08 (1.05–1.12) > 4 h/day). Associations with cancer mortality (1.03 (1.02–1.04)) and T2D were linear (1.09 (1.07–1.12)). *Conclusions:* Independent of PA, total sitting and TV viewing time are associated with greater risk for several major chronic disease outcomes. For all-cause and CVD mortality, a threshold of 6–8 h/day of total sitting and 3–4 h/day of TV viewing was identified, above which the risk is increased.

**Electronic supplementary material:**

The online version of this article (10.1007/s10654-018-0380-1) contains supplementary material, which is available to authorized users.

## Introduction

Since the mid-twentieth century people have spent an increasingly greater amount of their time sedentary [1, 2]. Sedentary behaviours are defined as any waking time activity during which one is in seated, reclined or lying posture, expending low levels of energy [3, 4]. Americans spend 55% of their waking time, or 7.7 h/day, sedentary [5]. Europeans are estimated to spend on average 40% of their leisure time watching TV [6], equal to 2.8 h/day in the UK, which is not declining [7]. Accumulation of sedentary time is independent from lack of accumulation of moderate-to-vigorous physical activity (MVPA), e.g. sufficient levels in MVPA do not preclude relatively high levels of sedentary time and vice versa [8–10]. Moreover, the health effects of sedentary behaviours tend to persist, with some attenuation, after accounting for MVPA [11–13]. One recent meta-analysis including over 1 million adults documented that high levels of sitting time increased premature mortality risk in all but the most physically active individuals who accumulate ≥ 1 h/day of moderate-intensity activity [12].

Previous meta-analyses have attempted to estimate the potential impact of sedentary behaviour on specific health outcomes [9–12, 14–17]. However, they were not without considerable limitations, such as inclusion of non-prospective studies [10, 11] and use of an ambiguous sedentary behaviour exposure, defined by different exposure types across studies (i.e. a mix of total sitting, TV viewing or total leisure sitting time, which show different health associations [10–12]), and/or different exposure units or categories [10, 11, 16, 17]. Most importantly, few meta-analyses have examined dose–response associations, to determine whether there is a marked increase in risk of incident disease or mortality at a specific level on the sedentary time continuum [12, 14–17]. This information is essential to determine whether recommendations, currently only providing guidance to “sit less”, need further quantification. For all-cause mortality, spending > 3 or > 4 h/day of TV viewing and > 7 h/day in any sitting activity have been suggested as detrimental [14–16]. It is currently unknown whether these thresholds (if any) are the same for cardiovascular disease (CVD) and cancer mortality. For type 2 diabetes (T2D), existence of such threshold has only been examined in relation to TV viewing time (based on 3 studies only) [15], which is not reflective of total sitting time. Recent studies reported 3.8–5.9% of all deaths are due to daily sitting time [14, 18]. So far this is unknown for TV viewing time, which shows stronger health associations [12] and may be one of the most amenable types of sedentary behaviour [19].

We therefore aimed to examine the dose–response association between separate types of sedentary behaviour and all-cause, CVD and cancer mortality, as well as incident T2D and CVD, using the current prospective evidence. As PA is known to attenuate sedentary behaviour associations [12], we also aimed to map this attenuating effect across the whole continuous sedentary behaviour dose-spectrum, by comparing dose–response curves with and without adjustment for PA. Finally, in order to demonstrate the population impact of the established dose–response relationships, we calculated the population attributable fraction (PAF) due to TV viewing for these health outcomes in England.

## Methods

### Data sources and searches

Studies had to have assessed the association between total daily sitting/sedentary, TV viewing or leisure sitting time, and at least one of the outcomes of interest: all-cause, CVD or cancer mortality, incident (fatal and non-fatal) CVD and incident T2D. Time spent sitting/sedentary could be self-reported or objectively measured. Only primary research studies with a prospective design, with at least an abstract in English and investigating non-diseased adults (≥ 18 years) in the general population were included.

Sources included:

1. Electronic literature databases: Pubmed, Web of Knowledge, Medline, Embase, Cochrane Library and Google Scholar from 1st August 2014 to 30th September 2016. Search terms are listed in Online Appendix Table 1.
2. Reference lists of existing systematic reviews [9–12, 14–17, 20, 21], examining associations between sedentary behaviours and health outcomes, which together cover up to October 2015.
3. Authors’ personal literature databases up to 30th September 2016.
4. Reference lists of included articles.

### Study selection

Titles and abstracts were screened by one author (RP) using the inclusion criteria, full reports were assessed where these were met or where there was uncertainty, allowing a final decision on eligibility. Where different articles formed part of the same cohort study, only data from the most recent publication for any given exposure-outcome combination was used. A minimum of 4 different eligible cohorts were required in order to carry out an analysis. Authors were contacted for additional information where needed.

### Data extraction and quality assessment

Using a pre-designed data extraction spreadsheet, two authors carried out independent extractions and disagreement was resolved through discussion (RP and EM).

The quality of each study was assessed using these criteria: size of cohort, length of follow-up, description of inclusion criteria and sampling strategy and sample representativeness, based on the Newcastle–Ottawa scale [22]. No overall quality score was assigned for use in analyses, to prevent the scale itself becoming a source of bias [23].

### Data synthesis and analysis

Extracted data were harmonized, converting each measure into one of: total sedentary, TV viewing or leisure sedentary time, quantified in h/day. Categories of sedentary time were assigned a dose, either the mid-point, or, in case of open-ended categories, half the width of the adjacent interval from the boundary (Online Appendix Tables 2 and 3). Where the lowest exposure was not the referent category, hazards were recalculated [24, 25].

Estimates of the linear association for each contributing study were calculated using Generalized Least-Squares regression [26, 27]. These were used to perform a random effects meta-analysis within each exposure-outcome combination, for both the most adjusted model without adjustment for PA and the least adjusted model with adjustment for PA [28]. These provided the summary RR per additional hour/day of exposure. Statistical heterogeneity of contributing studies was assessed with the I^2^ statistic, which was considered low if < 25%, and high if > 75% [29]. To examine publication bias and small study effect, Funnel plots were used, Egger’s tests were derived for each exposure-outcome combination with ≥ 5 contributing studies [30].

Following the estimation of a linear association, a restricted cubic spline transformation was carried out to investigate the shape of the dose–response relationship. Knots were placed at the 10th, 50th and 90th percentiles [31]. A random effects meta-analysis was then carried out to estimate the non-linear relationship between sedentary time and the respective health outcome. Where a change in strength of association was seen at a certain level on the exposure continuum, the RR (per h/day increment in exposure) on either side of this exposure level was calculated as the difference in log(RR) divided by difference in hours of exposure. Results were presented back on the original scale and were based on PA-adjusted analyses.

Sensitivity analyses were carried out to investigate the influence of study characteristics which might lead to risk of bias. Where sufficient data were available these were carried out on the linear PA adjusted associations. Factors investigated include: adiposity adjustment, sex, length of follow-up, age of cohort at baseline and representativeness of cohort.

### Population attributable fraction (PAF)

PAF estimates were calculated for TV viewing, indicating the proportional reduction in incidence of the respective outcome if prevalent TV viewing levels were reduced to zero, assuming causality. As we did not have access to TV viewing data worldwide, TV viewing data from the 2012 Health Survey for England (HSE), a nationally representative sample of the English population, were used to carry out a Monte-Carlo micro-simulation as an illustration of the potential magnitude of the impact. Each 17+ year old participant in HSE was probabilistically assigned an RR based on the RR and associated uncertainty from the PA-adjusted dose–response analysis which corresponded to their TV viewing category (0, 0 to < 2, 2 to < 4, 4 to < 6 and 6+ h/day of TV viewing). These assigned RRs, along with the proportional contribution of each individual, according to HSE survey weights, were used to calculate an attributable fraction for each participant, according to the formula below [32].

Equation 1—Population attributable fraction

1 $$PAF = \frac{{\sum\nolimits_{i = 1}^{n} {P_{i} RR_{i} - \sum\nolimits_{i = 1}^{n} {P_{i}^{\prime } RR_{i} } } }}{{\sum\nolimits_{i = 1}^{n} {P_{i} RR_{i} } }}$$

P_i_ = proportion of population at exposure level i, P_i_′ = proportion of population at counterfactual exposure level, i.e. zero exposure, RR = the relative risk at exposure level i. n = the number of exposure levels.

This procedure was then repeated 5000 times and the final PAF estimate was the median value across the 5000 simulations. A 95% CI was calculated using the 2.5th and 97.5th percentiles of the simulated distribution.

Stata version 14.2, StataCorp, USA was used for the meta-analysis. The PAF calculations were carried out using Analytica Free 101 edition, Lumina Decision Systems Inc., USA.

### Role of the funding source

This work was supported by the British Heart Foundation, the Medical Research Council, Cancer Research UK, Economic and Social Research Council, National Institute for Health Research, and the Wellcome Trust. The funders had no role in study design, conduct, or reporting of the results.

## Results

### Search results

**Fig. 1:**
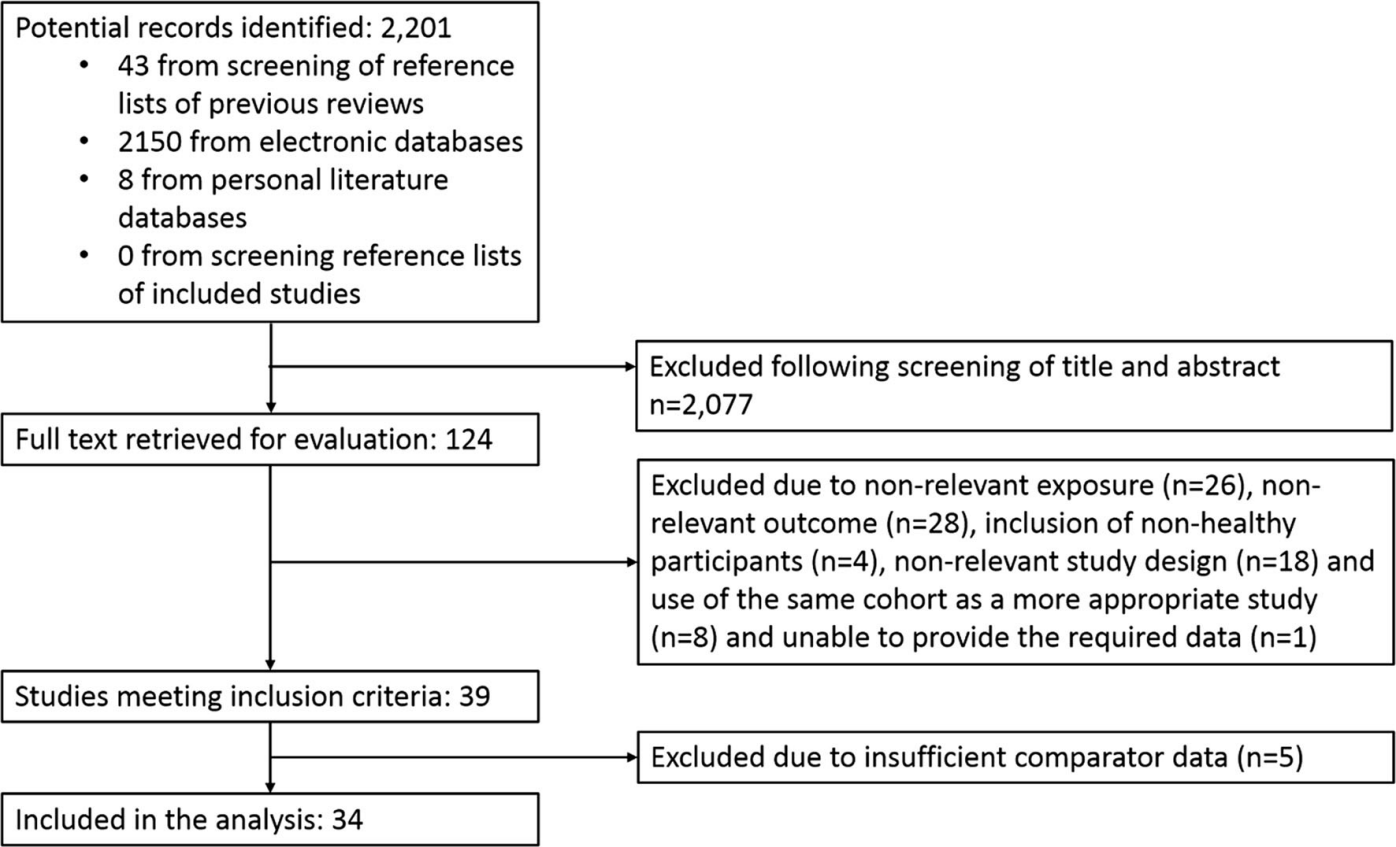
Flow diagram for study inclusion

The literature search provided 2201 potential articles. Following screening of titles and abstracts, full text was retrieved for 124 publications, rendering 39 studies for which inclusion criteria were met (Fig. 1). For 5 studies there was an insufficient number of comparator cohorts within the same exposure-outcome combination [33–37], leaving 34 studies, across 8 exposure-outcome combinations, to be included in the analysis [38–71]. An insufficient number of cohorts was identified to allow investigation of associations between leisure sedentary time with any outcome. There were also insufficient studies investigating incident CVD with any exposure. We were therefore unable to carry out the planned analysis on these exposures and outcomes. Additional data were successfully obtained from 11 studies [38–48].

### Study characteristics

Data from a total of 1,331,468 unique participants was included. Table 1 and Online Appendix Table 4 summarize the characteristics of the 34 included studies (with additional data from publications describing cohort characteristics [72–85]). The size of included studies ranged from 208 [51] to 240,819 [61] participants with a mean of 39,161. Follow-up was on average 8.9 years and varied from 2 [69] to 31 [46] years. The numbers of cases and participants by outcome are shown in Table 2. Of the 34 studies, 17 were from North America, 9 from Europe, 4 from Australia and 4 from Asia. The dates of publication spanned 2001–2016, although 65% were published in 2013 or later.

**Table 1:** Descriptive information on the 34 studies included in the analysis by outcome (studies for each outcome are sorted by the exposures investigated and first author’s name)

Authors	Country/cohort	Exposures investigated (events)	n at baseline/average follow-up (years)	Average age at baseline^a^/proportion male	Full list of covariates included in models
*All*-*cause mortality*					
Chau et al. [40]	Norway/HUNT3	Total sitting (640), TV viewing (684)	42,077/3.3	53.3/0.45	Sex, body mass index, education level, meeting PA guidelines, smoking status, general health status, cardio-metabolic disease (CMD) status with age as the time axis
Kim et al. [45]	USA/Multiethnic Cohort Study (MEC)	Total sitting (19,143), TV viewing (19,143)	134,596/13.7	59.0/0.46	Hazard ratios were calculated with age as the time metric, adjusted for age at cohort entry (5-year age groups), education, ethnicity and smoking history [including the following variables: smoking status, average number of cigarettes, average number of cigarettes squared, number of years smoked (time dependent), number of years since quitting (time dependent) and interactions between ethnicity and the smoking variables]. also adjusted for history of hypertension and/or diabetes at enrolment, alcohol consumption, energy intake and physical activity (METs per week for moderate activity, vigorous work and strenuous sports)
Pulsford et al. [66]	UK/Whitehall 2	Total sitting (450), TV viewing (324)	5132/15.7	43.9/0.73	Age, gender, employment grade and ethnicity, smoking status, alcohol consumption, fruit and vegetable consumption, BMI, physical functioning, daily walking time and MVPA.
Ensrud et al. [42]	USA/Osteoporotic Fractures in Men Study (MrOS)	Total sitting—accelerometer measured (409)	2918/4.5	79/1.00	Age, race, site, season, education, marital status, health status, smoking, comorbidity burden, depressive symptoms, cognitive function, number of instrumental activity of daily living impairments, and % body fat., Physical Activity Scale for the Elderly, Gait Speed, and Time Spent Asleep
Fox et al. [51]	UK/OPAL	Total sitting—accelerometer measured (32)	208/4.2	78.1/0.51	Adjusting for age, gender, educational attainment, IMD, weight status, GP Management System and number of self-reported chronic illnesses at baseline; additionally adjusting for lower limb function
Inoue et al. [55]	Japan/Japan Public Health Center-based prospective study group	Total sitting (4564)	83,034/8.7	56.0/0.47	Age (5-year age categories), area (11 PHC areas), occupation (stratified, full-time agriculture/forestry/fishery, full-time salaried/self-employed/professional, multiple occupations, full-time housework/retired/unemployed), history of diabetes (Y/N), smoking status (never, past, current), alcohol intake status (almost none, occasional, regular), BMI (14 to < 20, 20 to < 27, ≥ 27), total energy intake (quintiles), heavy physical work or strenuous exercise (none, < 1 h, ≥ 1 h), walking or standing hours (< 1 h, 1–3 h, ≥ 3 h), and leisure-time sports or physical exercise (< day/week, 1–2 days/week, ≥ 3–4 days/week)
Katzmarzyk et al. [56]	Canada/Canada Fitness Survey	Total sitting (1832)	17,013/12.0	42.0/0.43	Age, sex (as a continuous variable), smoking (former, current, non-smoker), alcohol consumption (abstainer, < 10 drinks per month, 10–50 drinks per month, > 50 drink per month), leisure time physical activity (as a continuous variable, METh wk^−1^), and the Physical Activity Readiness Questionnaire (pass/fail/missing)
Matthews et al. [60]	USA/Southern Community Cohort Study	Total Sitting (5007)	64,304/6.4	69.9/0.45	Sex, source of enrollment (community health center or general population), educational level (< 9 years, 9–11 years, high school, some college, or beyond college), household income (< $15,000, $15,000–$24,999, $25,000–$49,999, or ≥ $50,000), cigarette smoking (never; former, < 1 pack/day; former, ≥ 1 pack/day; current, < 1 pack/day; or current, ≥ 1 pack/day), BMI(< 18.5, 18.5–24.9, 25–29.9, 30.0–34.9, or ≥ 35.0), sleep duration (< 7, 7–8, or ≥ 9 h/day), diabetes (Y/N), employment status (Y/N), and overall physical activity level (quartiles). (Age was used as the underlying time metric)
Matthews et al. [62]	USA/NIH-AARP Diet and Health Study	Total sitting (12,201)	154,614/6.8	69.8/0.52	Age, Education (< 12 years, high school graduate, some college, college graduate, unknown), Smoking history (never, stopped 10+, 5–9, 1–4 years ago, stopped < 1 year, current smoker, unknown), Sleep duration (< 4, 4, 5.9, 6, 7.9, 8, 9.9, 10+ h/day, unknown), Overall health (excellent, very good, good, fair, poor, unknown), and BMI (< 25, 25, 29.9, 30+ kg/m^2^, unknown) additionally adjusted for Overall physical activity (< 1, 1–1.9, 2–2.9, 3–3.9, 4+ h/day)
Pavey et al. [63]	Australia/Australian Longitudinal Study on Women’s Health	Total sitting (1364)	47,53/6.0	78.0/0.00	Age, education, marital status, area, smoking, alcohol consumption, BMI, PA
Petersen et al. [64]	Denmark/Danish Health Examination Survey	Total sitting (1074)	71,363/5.4	48.1/0.40	Age, sex, education, physical activity level in leisure time, smoking habits, body mass index, alcohol consumption, diabetes, and hypertension
Schmid et al. [67]	USA/National Health and Nutrition Examination Survey	Total sitting—accelerometer measured (112)	1677/2.9	67.0/0.49	Age, sex, education, ethnicity, smoking, light physical activity, alcohol consumption, history of diabetes, history of cardiovascular disease (coronary heart disease, congestive heart failure, stroke), history of cancer, and mobility limitations. Plus accelerometer measured MVPA
Seguin et al. [68]	USA/Women’s Health Initiative (WHI) Observational Study (OS) and Extension Study (ES).	Total sitting (13,316)	92,234/12.2	64.0/0.00	Age, race, physical activities, and physical function score adjusted
van der Ploeg et al. [71]	Australia/45 and up cohort	Total sitting (4405)	22,2497/2.8	62.1/0.48	Age, sex, educational level, marital status, urban or rural residence, physical activity, BMI, smoking status, self-rated health, and receiving help with daily tasks for a long-term illness or disability
Basterra-Gortari et al. [39]	Spain/The SUN Cohort	TV viewing (97)	13,284/8.2	37/0.38	Age (continuous), sex, smoking history (never, current, quit), total energy intake, Mediterranean diet adherence (continuous), baseline body mass index (continuous), physical activity (quartiles), computer use (continuous) and driving (continuous)
Dunstan et al. [49]	Australia/The Australian Diabetes, Obesity and Lifestyle Study (AusDiab)	TV viewing (284)	8800/6.6	50.47/0.44	Age, sex, smoking (current or ex-smoker), education (≥ 12 years), total energy intake, alcohol intake, Diet Quality Index, waist circumference, hypertension (blood pressure ≥ 140/90 mm Hg or antihypertensive medication use), total plasma cholesterol (mmol/L), HDL-C (mmol/L), serum triglycerides (mmol/L, log), lipid-lowering medication use, and glucose tolerance status and exercise time
Keadle et al. [57]	USA/NIH-AARP Diet and Health Study	TV viewing (36,590)	221,426/14.1	62.5/0.57	Age (years), sex, race (white, black, other, missing), education (< 12 years, high school graduate, some college, college graduate, missing), smoking history (never; quit, ≤ 20 cigarettes/day; quit, > 20 cigarettes/day; current, ≤ 20 cigarettes/day; current, > 20 cigarettes/day; unknown), MVPA (never or rarely, 1, 1–3, 4–7, Z7 h/week) and diet quality (quintiles), BMI categories (18.5 to < 25, 25 to < 30, 30–35 and > 35 kg/m^2^) and health status (good, very good, excellent)
Matthews et al. [61]	USA/NIH-AARP Diet and Health Study	TV viewing (17,044)	240,819/8.5	62.5/0.56	Age, sex, race (white, black, other, missing), education (< 12 year, high school graduate, some college, college graduate, missing), smoking history (never; quit, ≤ 20 cigarettes/day; quit, > 20 cigarettes/day; current, ≤ 20 cigarettes/day; current, > 20 cigarettes/day; unknown), diet quality (quintiles) moderate-vigorous physical activity (never/rarely; < 1, 1–3, 4–7, > 7 h/week)
Muennig et al. [46]	USA/General Socal Survey	TV viewing (2048)	9344/^b^	44.0/0.42	Year, race, sex, education status, age, and income
Suzuki et al. [70]	Japan/Japan Collaborative cohort study	TV viewing (9880)	110,792/^b^	57.8/0.41	Age and area of study
Wijndaele et al. [48]	UK/EPIC-Norfolk	TV viewing (1270)	13,197/9.5	61.5/0.43	Age and gender, education level, smoking status, alcohol consumption, medication for hypertension (not in models examining cancer mortality), medication for dyslipidaemia (not in models examining cancer mortality), baseline history of diabetes, family history of CVD and family history of cancer, total PAEE (METh/day)
*CVD mortality*					
Kim et al. [45]	USA/Multiethnic Cohort Study (MEC)	Total sitting (6535), TV viewing (6535)	134,596/13.7	59.0/0.46	Hazard ratios were calculated with age as the time metric, adjusted for age at cohort entry (5–year age groups), education, ethnicity and smoking history [including the following variables: smoking status, average number of cigarettes, average number of cigarettes squared, number of years smoked (time dependent), number of years since quitting (time dependent) and interactions between ethnicity and the smoking variables]. also adjusted for history of hypertension and/or diabetes at enrolment, alcohol consumption, energy intake and physical activity (METs per week for moderate activity, vigorous work and strenuous sports)
Ensrud et al. [42]	USA/Osteoporotic Fractures in Men Study (MrOS)	Total sitting– accelerometer measured (138)	2918/4.5	79/1.00	Age, race, site, season, education, marital status, health status, smoking, comorbidity burden, depressive symptoms, cognitive function, number of instrumental activity of daily living impairments, and % body fat
Katzmarzyk et al. [56]	Canada/Canada Fitness Survey	Total sitting (759)	17,013/12.0	42.0/0.43	Age, sex (as a continuous variable), smoking (former, current, non-smoker), alcohol consumption (abstainer, < 10 drinks per month, 10–50 drinks per month, > 50 drink per month), leisure time physical activity (as a continuous variable, METh week^−1^), and the Physical Activity Readiness Questionnaire (pass/fail/missing)
Matthews et al. [60]	USA/Southern Community Cohort Study	Total Sitting (1376)	64,304/6.4	69.9/0.45	Sex, source of enrollment (community health center or general population), educational level (< 9 years, 9–11 years, high school, some college, or beyond college), household income (< $15,000, $15,000–$24,999, $25,000–$49,999, or ≥ $50,000), cigarette smoking (never; former, < 1 pack/day; former, ≥ 1 pack/day; current, < 1 pack/day; or current, ≥ 1 pack/day), BMI(< 18.5, 18.5–24.9, 25–29.9, 30.0–34.9, or ≥ 35.0), sleep duration (< 7, 7–8, or ≥ 9 h/day), diabetes (Y/N), employment status (Y/N), and overall physical activity level (quartiles) (age was used as the underlying time metric)
Matthews et al. [62]	USA/NIH-AARP Diet and Health Study	Total sitting (3339)	154,614/6.8	69.8/0.52	Age, Education (< 12 years, high school graduate, some college, college graduate, unknown), Smoking history (never, stopped 10+, 5–9, 1–4 years ago, stopped < 1 year, current smoker, unknown), Sleep duration (< 4, 4, 5.9, 6, 7.9, 8, 9.9, 10+ h/day, unknown), Overall health (excellent, very good, good, fair, poor, unknown), and BMI (< 25, 25, 29.9, 30+ kg/m^2^, unknown) additionally adjusted for Overall physical activity (< 1, 1–1.9, 2–2.9, 3–3.9, 4+ h/day)
Seguin et al. [68]	USA/Women’s Health Initiative (WHI) Observational Study (OS) and Extension Study (ES).	Total sitting (3878)	92,234/12.2	64.0/0.00	Age, race, physical activities, and physical function score adjusted
Dunstan et al. [49]	Australia/The Australian Diabetes, Obesity and Lifestyle Study (AusDiab)	TV viewing (87)	8800/6.6	50.47/0.44	Age, sex, smoking (current or ex-smoker), education (≥ 12 years), total energy intake, alcohol intake, Diet Quality Index, waist circumference, hypertension (blood pressure ≥ 140/90 mm Hg or antihypertensive medication use), total plasma cholesterol (mmol/L), HDL-C (mmol/L), serum triglycerides (mmol/L, log), lipid-lowering medication use, and glucose tolerance status and exercise time
Ikehara et al. [54]	Japan/The Japan Collaborative Cohort Study	TV viewing (5835)	85,899/19.2	41.9/0.42	Age and Sex, BMI, smoking, ethanol intake, education level, hours of sport, hours of walking, sleep duration, perceived mental stress, presence of job, frequency of fresh fish intake and depressive symptoms
Matthews et al. [61]	USA/NIH-AARP Diet and Health Study	TV viewing (4684)	240,819/8.5	62.5/0.56	Age, sex, race (white, black, other, missing), education (< 12 year, high school graduate, some college, college graduate, missing), smoking history (never; quit, ≤ 20 cigarettes/day; quit, > 20 cigarettes/day; current, ≤ 20 cigarettes/day; current, > 20 cigarettes/day; unknown), diet quality (quintiles) moderate-vigorous physical activity (never/rarely; < 1, 1–3, 4–7, > 7 h/week)
Warren et al. [47]	USA/ACLS Cohort	TV viewing (377)	7744/^b^	47.1/1.00	Age, physically inactive, current smoker, alcohol intake (less than one, between one and two, and more than two drinks per day), BMI, family history of CVD, hypertension, diabetes, and hypercholesterolemia
Wijndaele et al. [48]	UK/EPIC-Norfolk	TV viewing (373)	13,197/9.5	61.5/0.43	Age and gender, education level, smoking status, alcohol consumption, medication for hypertension (not in models examining cancer mortality), medication for dyslipidaemia (not in models examining cancer mortality), baseline history of diabetes, family history of CVD and family history of cancer, total PAEE (MET_h/day)
*Cancer mortality*					
Kim et al. [45]	USA/Multiethnic Cohort Study (MEC)	Total sitting (6697), TV viewing (6697)	134,596/13.7	59.0/0.46	Hazard ratios were calculated with age as the time metric, adjusted for age at cohort entry (5–year age groups), education, ethnicity and smoking history [including the following variables: smoking status, average number of cigarettes, average number of cigarettes squared, number of years smoked (time dependent), number of years since quitting (time dependent) and interactions between ethnicity and the smoking variables]. also adjusted for history of hypertension and/or diabetes at enrolment, alcohol consumption, energy intake and physical activity (METs per week for moderate activity, vigorous work and strenuous sports)
Ensrud et al. [42]	USA/Osteoporotic Fractures in Men Study (MrOS)	Total sitting– accelerometer measured (129)	2918/4.5	79/1.00	Age, race, site, season, education, marital status, health status, smoking, comorbidity burden, depressive symptoms, cognitive function, number of instrumental activity of daily living impairments, % body fat
Katzmarzyk et al. [56]	Canada/Canada Fitness Survey	Total sitting (547)	17,013/12.0	42.0/0.43	Age, sex (as a continuous variable), smoking (former, current, non-smoker), alcohol consumption (abstainer, < 10 drinks per month, 10–50 drinks per month, > 50 drink per month), leisure time physical activity (as a continuous variable, METh wk^−1^), and the Physical Activity Readiness Questionnaire (pass/fail/missing)
Matthews et al. [60]	USA/Southern Community Cohort Study	Total Sitting (1227)	64,304/6.4	69.9/0.45	Sex, source of enrollment (community health center or general population), educational level (< 9 years, 9–11 years, high school, some college, or beyond college), household income (< $15,000, $15,000–$24,999, $25,000–$49,999, or ≥ $50,000), cigarette smoking (never; former, < 1 pack/day; former, ≥ 1 pack/day; current, < 1 pack/day; or current, ≥ 1 pack/day), BMI(< 18.5, 18.5–24.9, 25–29.9, 30.0–34.9, or ≥ 35.0), sleep duration (< 7, 7–8, or ≥ 9 h/day), diabetes (Y/N), employment status (Y/N), and overall physical activity level (quartiles). (Age was used as the underlying time metric)
Matthews et al. [62]	USA/NIH-AARP Diet and Health Study	Total sitting (4507)	154,614/6.8	69.8/0.52	Age, Education (< 12 yrs, high school graduate, some college, college graduate, unknown), Smoking history (never, stopped 10+, 5–9, 1–4 years ago, stopped < 1 year, current smoker, unknown), Sleep duration (< 4, 4, 5.9, 6, 7.9, 8, 9.9, 10+ h/day, unknown), Overall health (excellent, very good, good, fair, poor, unknown), and BMI (< 25, 25, 29.9, 30+ kg/m^2^, unknown) additionally adjusted for Overall physical activity (< 1, 1–1.9, 2–2.9, 3–3.9, 4+ h/day)
Seguin et al. [68]	USA/Women’s Health Initiative (WHI) Observational Study (OS) and Extension Study (ES).	Total sitting (4759)	92,234/12.2	64.0/0.00	Age, race, physical activities, and physical function score adjusted
Dunstan et al. [49]	Australia/The Australian Diabetes, Obesity and Lifestyle Study (AusDiab)	TV viewing (125)	8800/6.6	50.47/0.44	Age, sex, smoking (current or ex-smoker), education (≥ 12 years), total energy intake, alcohol intake, Diet Quality Index, waist circumference, hypertension (blood pressure ≥ 140/90 mm Hg or antihypertensive medication use), total plasma cholesterol (mmol/L), HDL-C (mmol/L), serum triglycerides (mmol/L, log), lipid-lowering medication use, and glucose tolerance status and exercise time
Keadle et al. [57]	USA/NIH-AARP Diet and Health Study	TV viewing (15,161)	221,426/14.1	62.5/0.57	Age (years), sex, race (white, black, other, missing), education (< 12 years, high school graduate, some college, college graduate, missing), smoking history (never; quit, ≤ 20 cigarettes/day; quit, > 20 cigarettes/day; current, ≤ 20 cigarettes/day; current, > 20 cigarettes/day; unknown), MVPA (never or rarely, 1, 1–3, 4–7, Z7 h/week) and diet quality (quintiles), BMI categories (18.5 to < 25, 25 to < 30, 30–35 and > 35 kg/m^2^) and health status (good, very good, excellent)
Matthews et al. [61]	USA/NIH-AARP Diet and Health Study	TV viewing (7652)	240,819/8.5	62.5/0.56	Age, sex, race (white, black, other, missing), education (< 12 year, high school graduate, some college, college graduate, missing), smoking history (never; quit, ≤ 20 cigarettes/day; quit, > 20 cigarettes/day; current, ≤ 20 cigarettes/day; current, > 20 cigarettes/day; unknown), diet quality (quintiles) moderate-vigorous physical activity (never/rarely; < 1, 1–3, 4–7, > 7 h/week)
Suzuki et al. [70]	Japan/Japan Collaborative cohort study	TV viewing (3787)	110,792/^b^	57.8/0.41	Age and area of study
Wijndaele et al. [48]	UK/EPIC-Norfolk	TV viewing (570)	13,197/9.5	61.5/0.43	Age and gender, education level, smoking status, alcohol consumption, medication for hypertension (not in models examining cancer mortality), medication for dyslipidaemia (not in models examining cancer mortality), baseline history of diabetes, family history of CVD and family history of cancer, total PAEE (MET_h/day)
*Incident type 2 diabetes*					
Ding et al. [41]	Australia/45 and up	Total sitting (829)	54,997/3.4	45 +/0.46	Sex, age, quintile of socioeconomic disadvantage, household income categories, country of birth (categorised), highest education, CVD disease (yes/no), high cholesterol (yes/no), family history of T2DM (yes/no), smoking (yes/no), alcohol consumption ≤ drinks, > 14 drinks, total minutes of MVPA (≥ 300 min/150 to < 300 min/< 150 min), fruit intake per day (≥ 2 servings/< 2 servings), vegetable intake per day (≥ 5/< 5 servings), body weight status (healthy weight/underweight/overweight/obese) and psychological distress (none or low or moderate/high or very high)
Gibbs et at [43]	USA/The Coronary Artery Risk Development in Young Adults (CARDIA) Study	Total sitting (201)	1869/5	45.3/0.43	Adjusted for age, center, race, sex, education, income, smoking, alcohol, wear time, and log-transformed MVPA
Manini et al. [59]	USA/Women’s Health Initiative (WHI) Observational Study (OS)	Total sitting (7426)	88,250/11.1	63.95/0.00	Age, ethnicity and race, college education, income less than $35,000 per year, marital status, comorbidity propensity score (feeling depressed, hypertension, hyperlipidemia, osteoarthritis, history of cancer, and CVD), number of immediate family members with history of diabetes, currently smoking, alcohol intake > 7 drinks per week, percent of daily caloric intake as carbohydrate and percent of daily caloric intake as fat, BMI (kg = m2) and minutes performing MVPA
Petersen et al. [65]	Denmark/DANHES	Total sitting (1790)	72,608/4.9	48.5/0.40	Sex, age, education (< 12, 12–14 and 15+ years), smoking habits(never-smoker, ex-smoker, occasional smoker, daily smoker (1–15 g of tobacco/day) and heavy smoker (> 15 g of tobacco/day)), body mass index (continuous), alcohol consumption (number of drinks/week), and hypertension (yes current/yes previously, no) and previous cardiovascular disease (yes current/yes previously, no) and MVPA (summed from 4 domains (work, transport, domestic and leisure time using IPAQ)
Anjana et al. [38]	India/Chennai urban rural epidemiology study (CURES-142)	TV viewing (385)	1376/10	38/0.42	Age, gender, family history of diabetes, physical inactivity, generalized obesity, abdominal obesity, household income, total energy, energy adjusted saturated fatty acid (SFA g/day in quartiles), and dietary fiber (g/day in quartiles) for the selected diet variables. In addition, added sugars (g/day) and meat intake (g/day) were further adjusted for dairy intake
Ford et al. [50]	Germany/EPIC–Potsdam	TV viewing (927)	23,855/7.8	49.7/0.38	Age, sex, educational status, and occupational activity, plus smoking status, alcohol use, and physical activity
Hu et al. [52]	USA/The health professional follow-up study (HPFS)	TV viewing (767)	31,379/8.0	55.3/1.00	Age, length of follow up, smoking, parental history of diabetes, alcohol consumption, and physical activity
Hu et al. [53]	USA/Nurses’ Health Study	TV viewing (1515)	68,497/5.3	57.4/0.00	Age, hormone use, alcohol consumption, smoking, family history of diabetes, and physical activity
Joseph et al. [44]	USA/Multi-ethnic study of atherosclerosis (MESA)	TV viewing (655)	5829/11.1	61.8/46.4	Age, race, gender, education, current occupation status, study site, current smoking, systolic blood pressure, and current hypertension medication usage
Krishnan et al. [58]	USA/The Black Women’s Health Study (BWHS)	TV viewing (2928)	45,668/4.0	21–69/0.00	Age, time period, family history of diabetes, years of education, family income, marital status, smoking, alcohol consumption, energy intake, coffee consumption, vigorous activity television watching and walking
Smith et al. [69]	UK/English Longitudinal Study of Aging—ELSA	TV viewing (129)	5964/2.0	64.6/0.44	Age, sex, physical activity, smoking, alcohol, depressive symptoms, long-standing illness, disability (impairment in activities of daily living/instrumental activities of daily living)

^a^Where a range is presented this was age range of included participants

^b^Information not available

**Table 2:** Summary linear estimates of associations between sedentary behaviour (hours/day) with major chronic disease outcomes from random-effects meta-analysis

Outcome (cases/n baseline)	Exposure (number of contributing studies for non-PA adjusted/PA adjusted estimates)	Without PA adjustment				With PA adjustment			
		Summary RR (95%CI)	*p* value RR = 1	I^2^ (%)	*p* value non-linearity	Summary RR (95%CI)	*p* value RR = 1	I^2^ (%)	*p* value non-linearity
All-cause mortality (110,395/1,138,042)	Total sedentary behaviour (12/13)	1.03 (1.02, 1.04)	< 0.001	49.7	< 0.001	1.02 (1.01, 1.03)	< 0.001	65.6	< 0.001
	TV viewing (7/7)	1.07 (1.04, 1.09)	< 0.001	70.9	< 0.001	1.05 (1.04, 1.05)	< 0.001	0	< 0.001
CVD mortality (24,042/667,524)	Total sedentary behaviour (6/5)	1.03 (1.02, 1.03)	< 0.001	0	0.001	1.02 (1.01, 1.03)	0.004	22.7	0.010
	TV viewing (5/6)	1.05 (1.02, 1.09)	0.005	90.1	0.203	1.04 (1.01, 1.08)	0.028	88.8	< 0.001
Cancer mortality (35,241/684,673)	Total sedentary behaviour (6/5)	1.01 (1.00–1.02)	0.212	84.1	0.168	1.01 (1.00, 1.02)	0.268	77.3	0.326
	TV viewing (4/4)	1.03 (1.02, 1.04)	< 0.001	0	0.642	1.02 (1.01, 1.03)	< 0.001	0	0.648
Type 2 diabetes (17,552/400,292)	Total sedentary behaviour (4)	[Insufficient number of studies]				1.01 (1.00, 1.01)	< 0.001	0	0.529
	TV viewing (5/6)	1.12 (1.08, 1.16)	< 0.001	65.9	0.767	1.09 (1.07, 1.12)	< 0.001	31.7	0.066

### Study quality and methods of measurement

The quality of the included studies was generally good (see Table 1 and Online Appendix Table 4). Three of the five smallest cohorts (presenting 32 cases/208 participants [51], 112/1677 [67], and 409/2918 [42]) measured sedentary time objectively. Of 34 studies, 22 had ≥ 10,000 participants [39–41, 45, 48, 50, 52–62, 64, 65, 68, 70, 71]. There were 3 all-male studies [42, 47, 52] and 5 all-female studies [53, 58, 59, 63, 68] (providing data from 4 cohorts). Some articles presented results for multiple exposures and/or outcomes, with 34 publications presenting 57 analyses; the numbers of contributing articles for each exposure/outcome combination are presented in Table 2.

Most studies assessed sedentary behaviour by questionnaire (31 out of 34) with three studies measuring sedentary time objectively using accelerometers worn on the participant’s hip, waist or lower back for up to 7 days (Online Appendix Table 4) [42, 51, 67].

Outcomes were assessed objectively in 27 of the 34 studies (Online Appendix Table 4). This represents those studies with a mortality outcome assessed using death registries, in addition to four studies which used an objective measure to define T2D status [38, 44, 50, 65], the remainder being self-reported T2D.

All included studies reported adjusted effect estimates, with adjustment for PA present in all but four studies [44, 46, 51, 70]. One study presented results for some outcomes with PA adjustment and some without [42]. Adjustment for PA varied in detail; from simply meeting the PA guideline or not, to calculating weight-adjusted energy expenditure across multiple domains of PA (Online Appendix Table 4).

### All-cause mortality

**Fig. 2:**
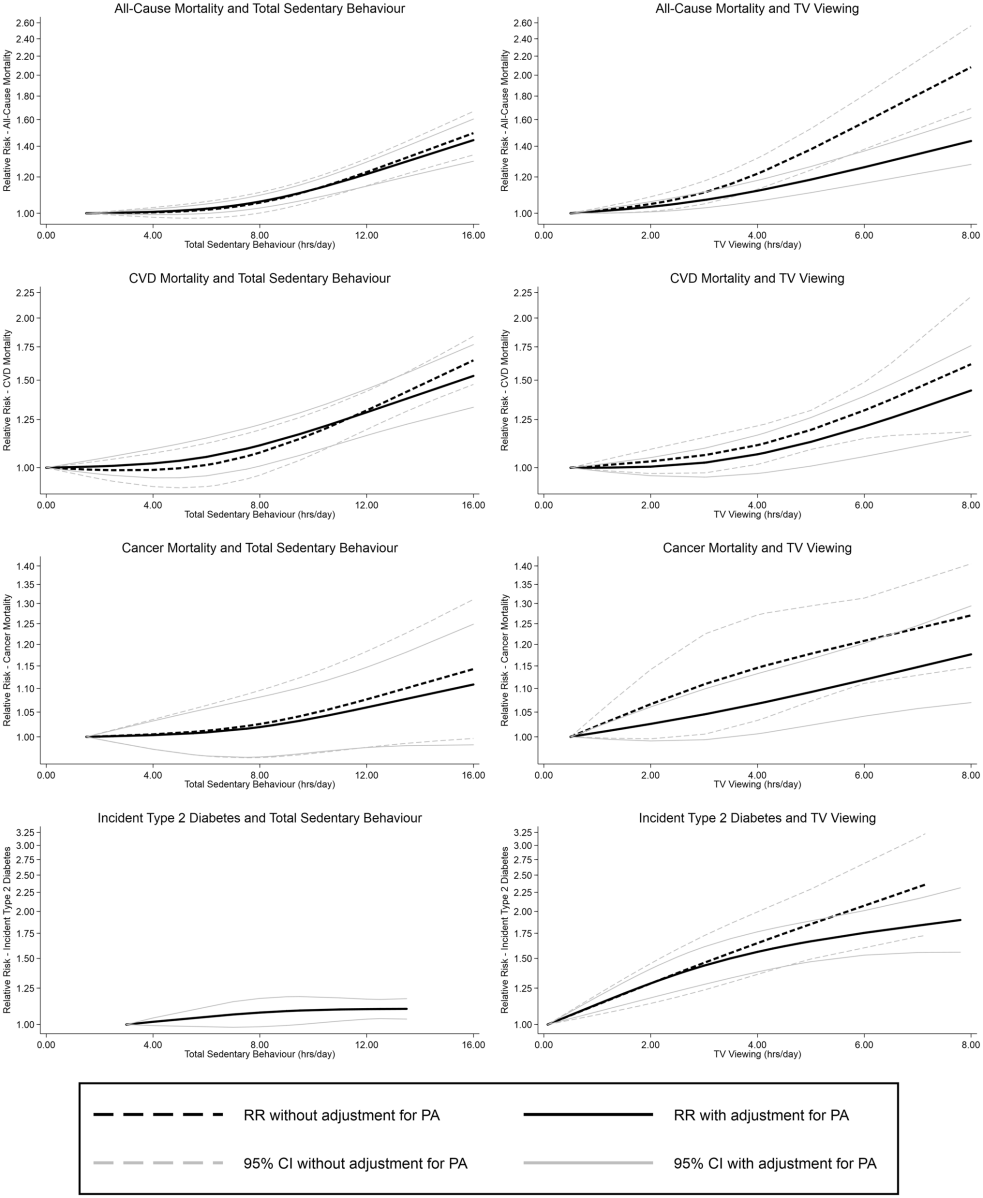
Non-linear associations between sedentary behaviour and health outcomes presented with and without PA adjustment

The association between total sedentary behaviour and all-cause mortality appeared to be non-linear, both with and without adjustment for PA (Fig. 2). Testing for non-linearity supported this finding. At lower levels of exposure, there were small increases in risk associated with increasing sedentary behaviour; above approximately 8 h/day of sedentary behaviour, the risk increased more rapidly. In PA adjusted analyses this resulted in an estimated RR of 1.01 (1.00–1.01) for each additional hour of exposure below 8 h/day and 1.04 (1.03–1.05) for each hour above 8 h/day (Online Appendix Table 5).

For TV viewing, the association also appeared to be non-linear (Fig. 2 and Table 2) with a change in gradient at approximately 3.5 h/day in the PA adjusted analyses (Fig. 2). Below this level the RR was 1.03 (1.01–1.04) per hour/day, and greater increases in risk were seen above this level (RR = 1.06 (1.05–1.08) per h/day; Online Appendix Table 5). Due to insufficient availability of data, investigation of the associations between leisure-time SB and mortality could not be undertaken.

### CVD mortality

For total sedentary behaviour, non-linearity was seen again for both the non-PA adjusted and PA adjusted models (Fig. 2 and Table 2). In the PA adjusted analysis, the threshold was in the region of 6 h/day of exposure, below which each additional hour was associated with an estimated RR of 1.01 (0.99–1.02) and above which each additional hour was associated with an RR of 1.04 (1.03–1.04) (Online Appendix Table 5).

The PA adjusted non-linear association between CVD mortality and TV viewing showed greater risk increases with every hour above a threshold of approximately 4 h/day (Fig. 2). The estimated RR for each additional hour of TV viewing was 1.02 (0.99–1.04) below 4 h and 1.08 (1.05–1.12) above.

### Cancer mortality

The linear association between total sedentary behaviour and cancer mortality was marginally non-significant and unaffected by PA adjustment; the PA adjusted estimate was 1.01 (1.00–1.02). There was no evidence for non-linearity (Table 2).

The linear association between TV viewing and cancer mortality was estimated to be 1.03 (1.02–1.04) in non-PA adjusted models and 1.02 (1.01–1.03) when adjusted for PA. There was no evidence for non-linearity for either PA adjusted or unadjusted associations (Fig. 2 and Table 2).

### Type 2 diabetes

The linear association between total sedentary behaviour and T2D was estimated to be 1.01 (1.00, 1.01), which could only be estimated with PA adjustment as there were insufficient studies without PA adjustment (Table 2 and Fig. 2).

PA adjusted analysis of the association between TV viewing and T2D shows some deviation from linearity (Fig. 2) although statistical evidence was equivocal (*p* = 0.066; Table 2). The PA adjusted linear association was estimated to be 1.09 (1.07, 1.12) However, larger increases in risk were seen with increasing TV viewing below approximately 4 h (1.12 (1.08–1.15) in PA adjusted analysis), above this level increasing TV viewing was associated with lower increases in risk (1.05 (1.03–1.07)). In non PA-adjusted analyses the association appeared linear with an estimated RR of 1.12 (1.08, 1.16) associated with each additional hour of TV viewing.

Across all combinations, PA adjustment appeared to attenuate the effect estimates. The difference between the effect sizes with and without PA adjustment was relatively small when total sedentary time was the exposure, but somewhat greater when TV viewing was examined. Substantial heterogeneity was observed for the pooled effect estimates, ranging from I^2^ values of 0% for both total sedentary behaviour and CVD, and total sedentary behaviour and cancer mortality, to I^2^ of 90.1% for TV viewing and CVD mortality. Funnel plots and Egger’s tests, showed no definitive evidence for publication bias (Online Appendix Fig. 5). However, the low numbers of contributing studies for some associations made it difficult to rule out these biases.

Sensitivity analyses are presented in Online Appendix Table 6. The RR of CVD mortality associated with each additional hour of TV viewing was greater when studies of younger participants were excluded, 1.08 (1.06–1.10) compared with 1.04 (1.01–1.08) in the main analysis. All other sensitivity analyses showed no substantive change from the main findings.

### Population attributable fractions for TV viewing

For all-cause mortality, 8% (6–10%) was associated with TV-viewing in the English population, when using the PAF method. This estimate was 5% (1–8%) for CVD and 5% (2–7%) for cancer mortality. For T2D 29% (26–32%) of incidence was estimated to be related to TV-viewing.

## Discussion

This meta-analysis, incorporating data of 1,331,468 participants, shows an increased risk for all-cause and CVD mortality and incidence of T2D with higher levels of total sitting as well as TV viewing time, independent of PA. For all outcomes, associations with TV viewing were stronger, and the strongest association overall was found between TV viewing and incident of T2D. There was also evidence of an independent association between sedentary behaviour and cancer mortality, although only for a specific type of sedentary behaviour, i.e. TV viewing time.

Most importantly, investigation of the shape of the associations indicated that the increased risk of all-cause and CVD mortality was strongest for sitting time volumes greater than 8 and 6 h/day, respectively, in PA adjusted analyses. For TV viewing time, an increased risk for all-cause and CVD mortality was strongest above levels of about 3–4 h/day. The associations between TV viewing with T2D and cancer mortality appeared to be more linear. In general, PA adjustment resulted in some attenuation of the estimated linear and non-linear associations, which was somewhat stronger for TV viewing compared to total sitting time. Furthermore, we estimated that a sizeable fraction of mortality and incidence of all examined outcomes were associated with TV viewing, ranging from 5% for CVD and cancer mortality, to a substantial 29% for T2D.

### Potential mechanisms

Biological mechanisms have been suggested to explain the independent associations of sedentary behaviour, in particular for cardio-metabolic diseases, through independent effects of prolonged sitting on lipid and glucose metabolism in the large skeletal muscles involved in posture (legs and core) and on hemodynamic vascular signalling potentially causing atherogenesis [86–89]. Associations for TV viewing were generally stronger than those for total sitting time with the same outcome which could be explained by several factors. Firstly, TV viewing has been linked to higher intakes of energy and macronutrients along with greater energy from snacks [90]. Poor diet quality and increased total calorie intake have been associated with increased risk of mortality and is a strong determinant of T2D, suggesting an important mediating role for dietary intake which is likely less relevant for total sitting time [91, 92]. Second, a potentially different confounding structure for TV viewing may be more difficult to fully account for. Third, criterion validity of self-reported TV viewing estimates tends to be stronger than those for total sitting time estimates [93, 94]. Lastly, the typical timing of TV viewing, i.e. in the evening following the main meal of the day [95], may exacerbate the repetitive cardiovascular effects of postprandial glucose and lipid excursions following this meal, especially if TV viewing is predominantly accumulated in prolonged bouts of sitting [96, 97].

### Limitations of the available evidence

The methods used to measure exposure varied; measurement of sedentary behaviour is still primarily reliant on self-report questionnaires. Heterogeneity in question phrasing, the time period considered and whether a question is single or multipart can all influence validity [93]. Misclassification of sedentary exposure would potentially dilute the association in our analysis, resulting in possible underestimation of effect size. The use of accelerometer measured sedentary time addresses some of the limitations of questionnaires, however this data has its own limitations. For example, some accelerometer methods cannot detect cycling or swimming, or fail to distinguish between sitting/lying and standing still [87]. This substantial heterogeneity in exposure measurement contributed to the high heterogeneity indices (I^2^) for the pooled estimates which may have influenced our overall findings. It is possible that only some of the constituents of total sitting are detrimental to health, for example sitting while reading is potentially advantageous [98]. That we were unable to investigate leisure sitting time in this meta-analysis due to insufficient studies would indicate that more research is required on the effect of different domains of sedentary behaviour. In addition to the exposure measure, the quality of the measurement of important covariates, such as PA, diet and socio-economic position, varied greatly between studies, if included at all, potentially leading to residual confounding. The low number of studies for some combinations meant that investigation of leisure time sitting could not be carried out. It also led to a lack of statistical power for subgroup or sensitivity analysis and bias assessment. This also meant that meta-regression techniques to investigate the impact of the potential sources of heterogeneity were precluded.

### Strengths and limitations of the meta-analysis

This meta-analysis considered total sedentary behaviour and TV viewing time as separate exposures. This is important as they have different associated socio-demographic and/or behavioural patterns (e.g. dietary intake) and therefore different confounding/mediating patterns [6, 90]. Inclusion of emerging research using objectively measured sedentary time is another strength. In addition, we investigated the shape of the dose–response curves, to identify where the greatest risk/benefits lie along the spectrum of exposure for all exposure—outcome combinations. Moreover, to our knowledge, this is the first study to calculate PAF estimates for TV viewing time and all considered health outcomes based on meta-analytical risk estimates when potential non-linearity of associations were taken into account.

However, our meta-analysis also has certain limitations. The use of summary data means heterogeneity of used statistical methods may influence comparability of included studies [29]. In order to investigate the effect of PA adjustment we had to select models which were as similar as possible except for adjustment for PA. However, in some studies additional differences in covariates were seen between these models and this may have resulted in residual confounding of the considered study-specific risk estimates. It was also necessary to make several assumptions during the dose-assignment procedure. Whilst these assumptions may have been crude in studies reporting little detail on the exposure, this approach allowed us to consider the totality of the currently published evidence. Treating the many and heterogeneous conditions that make up cancer as one outcome may have contributed to our mixed findings with these analyses. Investigating separate cancer types may be more informative, where there is enough data [99]. Attempting to reduce reverse causality by only including prospective studies may not have been entirely effective, especially in the case of T2D. An estimated 27% of those with the condition have no formal diagnosis, therefore having the condition may have preceded ascertainment of exposure data [100]. Finally, the calculation of PAFs rests on the assumption of causality, and the use of unbiased estimates with no measurement error.

### Public health impact

To calculate the PAF estimates we have used the exposure profile representative of the population of England. These might not be representative for other countries. However, average US TV viewing levels, for example, are similar, 2.6 h on a weekday and 3.3 h on a weekend day compared with 2.7 h and 3.1 h/day respectively in England [101]. The estimated 29% of T2D in England in 2012 that could be prevented or postponed by eliminating TV viewing, assuming causality, reflects the high RRs seen for this association. The linear association and relatively high RR even at lower exposure levels are important contributors, as a large proportion of HSE participants report lower TV viewing levels (75% of participants report < 4 h/day), but only a small proportion reports no TV viewing (3%). The PAF estimates for all-cause mortality (8%) and CVD and cancer mortality (both 5%) also suggest a potentially important burden of preventable disease from current population levels of TV viewing.

The differing nature of the relationships between TV viewing and different outcomes may complicate any prevention strategy. The prevention of T2D would perhaps be best served by reducing TV viewing among the whole population, however, to prevent other outcomes targeting those with highest exposure levels may be more appropriate as these are the individuals for whom any reduction would confer the greatest benefit. Furthermore, the effect of any behaviour change will also be influenced by the nature of the replacement activity [13]. For example, greater reductions in risk may occur when replacing sedentary time with strenuous exercise compared with walking for pleasure. Replacing some sedentary behaviours may confer greater benefits than others, e.g. replacing TV viewing may be more beneficial than replacing general screen time [13].

Another potentially important determinant of the health effects of sedentary behaviour is the extent to which breaks are taken in extended periods of sitting. None of the studies included in this meta-analysis took into account accumulation pattern of sitting and therefore this falls outwith the scope of this study.

## Conclusion

This study demonstrates an increasing risk of disease and mortality with increasing total sitting time and TV viewing time. It also revealed a threshold of 6–8 h/day of total sitting and 3–4 h/day of TV viewing, above which risk for several important health outcomes increased more rapidly. This suggests that sedentary behaviour guidelines may need further quantification of sitting time volumes that should be avoided, although for some outcomes such as T2D, any sitting time reductions would be beneficial. With 8% of all mortality and 29% of T2D in the English population associated with certain sedentary behaviours, there is great potential for substantial public health benefits. Improvements in the measurement of sedentary time and a better understanding of its confounding structure are therefore essential to improving future public health and clinical guidelines.

## Electronic supplementary material

Below is the link to the electronic supplementary material.

Supplementary material 1 (DOCX 264 kb)
