# Supplementary material for: Sedentary behaviour and risk of all-cause, cardiovascular and cancer mortality, and incident type 2 diabetes: a systematic review and dose response meta-analysis
Source: Eur J Epidemiol. 2018 Mar 28;33(9):811–29. doi: 10.1007/s10654-018-0380-1 (PMC6133005; doi:10.1007/s10654-018-0380-1)
Supplement: Supplementary file 1 — Supplementary material 1 (DOCX 264 kb) [file 10654_2018_380_MOESM1_ESM.docx]

**SUPPLEMENTAL MATERIAL**

**CONTENTS**

Appendix Table 1 – Search Terms

Appendix Figure 1 – Forest plot of the associations between sedentary behaviours and health outcome without adjustment for physical activity

Appendix Figure 2 – Forest plot of the associations between sedentary behaviours and health outcome with adjustment for physical activity

Appendix Table 4 – additional information about the quality of the 34 studies included in the analysis by outcome

Appendix Figure 3 – Non-linear associations between sedentary behaviours and health outcome without adjustment for physical activity

Appendix Figure 4 – Non-linear associations between sedentary behaviours and health outcome with adjustment for physical activity

Appendix Table 5. Relative risks (95% CI) below and above the exposure level corresponding with a change in strength of association, for all-cause and CVD mortality

Appendix Figure 5 - Funnel Plots with pseudo 95% confidence limits and Egger’s test for fully adjusted analyses with at least 5 contributing studies

Appendix table 6 - sensitivity analyses using linear PA adjusted associations

Appendix Table 7 - full output from the PA adjusted non-linear dose response meta-analysis for the associations of total sitting with health outcomes

Appendix Table 8 - full output from the PA adjusted non-linear dose response meta-analysis for the associations of TV viewing with health outcomes

Appendix Table 1 – Search Terms

Search terms for the electronic literature database search in: Pubmed, Web of Knowledge, Medline, Embase, Cochrane Library. Where possible the dates used were from 1^st^ August 2014 to 30th September 2016.

|  | sedentary OR sitting OR television OR TV OR screen time |
| --- | --- |
| AND | physical activity OR habitual physical activity OR MVPA |
| AND | mortality OR death OR fatal OR diabetes OR cardiovascular OR CVD OR cancer OR neoplasm OR carcinoma |
| AND | Risk OR Cox OR hazard OR survival OR odds |
| NOT | Child* OR youth OR adolescen* |
|  |  |

Google scholar differed significantly from the other search engines used, so an adapted search strategy was required, consisting of:

| - with the exact phrase: | “sedentary time” |
| --- | --- |
| - with at least one of: | “all-cause mortality” “cancer mortality” “cardiovascular mortality” “incident diabetes” “incident cardiovascular disease” |
| - without the words: | children youth adolescent |
| - words occur: | anywhere in the article |
| - Return articles dated between: | 2014-2016 |
|  |  |

Appendix Figure 1 – Forest plot of the linear RR for a 1 hour per day increase in sedentary behaviour and health outcome without adjustment for physical activity. Squares around point estimates represent weighting in meta-analysis.

Appendix Figure 2 – Forest plot of the linear RR for a 1 hour per day increase in sedentary behaviour and health outcome with adjustment for physical activity. Squares around point estimates represent weighting in meta-analysis.

1 Chau 2013(40), 2 Ensrud 2014(42), 3 Fox 2015(51), 4 Katzmarzyk 2009(56), 5 Kim 2013(45), 6 Matthews 2014(60), 7 Matthews 2015(62), 8 Pavey 2015(63), 9 Petersen 2014(64), 10 Pulsford 2015(66), 11 Schmid 2015(67), 12 Seguin 2014(68), 13 Basterra-Gortari 2014(39), 14 Dunstan 2010(49), 15 Matthews 2012(61), 16 Muennig 2013(46), 17 Suzuki 2007(70), 18 Wijndaele 2011(48), 19 Ikehara 2015(54), 20 Warren 2010(47), 21 Ford 2010(50), 22 Hu 2001(52), 23 Hu 2003(53), 24 Joseph 2016(44), 25 Smith 2014(69), 26 Inoue 2008(55), 27 van der Ploeg 2012(71), 28 Keadle 2015(57), 29 Ding 2015(41), 30 Gibbs 2015(43), 31 Manini 2014(59), 32 Peterson 2016(65), 33 Anjana 2015(38), 34 Krishnan 2009(58).- These dictate the order of the references in Endnote to match those in the Stata graphs. To blank out by making text white.

Appendix Table 2 – dose assignments for studies using total sedentary sitting/sedentary time

|  | | | | | | | | | | | | | |
| --- | --- | --- | --- | --- | --- | --- | --- | --- | --- | --- | --- | --- | --- |
|  |  | Categories used by study authors (hours/day unless otherwise stated) | | | | | | Dose applied to exposure category | | | | | |
|  | **Exposure** | **1** | **2** | **3** | **4** | **5** | **6** | **1** | **2** | **3** | **4** | **5** | **6** |
| Ensrud et al.(42) | Total sedentary time  (mins/day) | <772.2 | 772.2-844.6 | 844.7-914.9 | ≥915 |  |  | 6.44 | 13.47 | 14.66 | 15.84 |  |  |
| Fox et al.(51) | Total sedentary time  (mins/day) | <633 | 633-696 | >696 |  |  |  | 5.28 | 11.08 | 12.13 |  |  |  |
| Schmidl et al.(67) | Total sedentary time | <8.6 | >=8.6 |  |  |  |  | 4.3 | 12.9 |  |  |  |  |
| Petersen et al. (2014)(64) | Total sitting | 0-<6 | 6-<10 | 10+ |  |  |  | 3 | 8 | 12 |  |  |  |
| Chau et al.(40) | Total sitting | <4 | 4-<7 | 7-<10 | ≥10 |  |  | 2 | 5.5 | 8.5 | 11.5 |  |  |
| Ding et al.(41) | Total sitting | <8 | 8-<11 | >11 |  |  |  | 4 | 9.5 | 12.5 |  |  |  |
| Gibbs et al.(43) | Total sitting | <6 | 6-<8 | 8-<10 | ≥10 |  |  | 3 | 7 | 9 | 11 |  |  |
| Inoue et al.(55) | Total sitting | <3 | 3-8 | ≥8 |  |  |  | 1.5 | 5.5 | 10.5 |  |  |  |
| Katzmarzyk et al.(56) | Total Sitting | almost none of the time | ¼ of the time | ½ the time | ¾  of the time | almost all of the time |  | 1.5 | 4 | 8 | 12 | 16 |  |
| Manini et al.(59) | Total sitting | ≤7 | 8-11 | 12-15 | ≥16 |  |  | 3.5 | 9 | 13.5 | 18.5 |  |  |
| Matthews et al. (2014)(60) | Total sitting | <5.76 | 5.76-8.5 | 8.51-12 | >12 |  |  | 2.88 | 7.13 | 10.26 | 13.75 |  |  |
| Matthews et al. (2012)(61) | Total sitting | <3 | 3-4 | 5-6 | 7-8 | ≥9 |  | 1.5 | 4 | 6 | 8 | 10 |  |
| Pavey et al.(63) | Total sitting | 0-<4 | 4-<8 | 8-<11 | ≥11 |  |  | 2 | 6 | 9.5 | 12.5 |  |  |
| Peterson et al. (2016)(65) | Total sitting | 0-6 | 6-<10 | 10+ |  |  |  | 3 | 8 | 12 |  |  |  |
| Pulsford et al.(66) | Total sitting  (hours/week) | ≥0, <26 | ≥26, <41 | ≥41, <55 | ≥55 |  |  | 1.857 | 4.786 | 6.857 | 8.857 |  |  |
| Seguin et al.(68) | Total sitting | <4 | >4-8 | >8-11 | ≥11 |  |  | 2 | 6 | 9.5 | 12.5 |  |  |
| Van der Ploeg et al.(71) | Total sitting | 0-<4 | 4-<8 | 8-<11 | ≥11 |  |  | 2 | 6 | 9.5 | 12.5 |  |  |
| Kim et al.(45) | Total sitting | <5 | 5-<10 | ≥10 |  |  |  | 2.5 | 7.5 | 12.5 |  |  |  |
| Matthews et al. (2015)(62) | Total sitting | <5 | 5-6.9 | 7-8.9 | 9-11.9 | ≥12 |  | 2.5 | 6 | 8 | 10.5 | 13.5 |  |

Appendix Table 3 – Dose assignments for studies using TV viewing time

|  | | | | | | | | | | | | | |
| --- | --- | --- | --- | --- | --- | --- | --- | --- | --- | --- | --- | --- | --- |
|  | **Exposure** | Categories used by study authors (hours/day unless otherwise stated) | | | | | | Dose applied to exposure category | | | | | |
| Anjana et al.(38) | TV viewing | **1** | **2** | **3** | **4** | **5** | **6** | **1** | **2** | **3** | **4** | **5** | **6** |
| Basterra-gortari et al.(39) | TV viewing | <1 | 1-2 | 2-3 | ≥3 |  |  | 0.5 | 1.5 | 2.5 | 3.5 |  |  |
| Chau et al.(40) | TV viewing | <1 | 1-3 | ≥4 |  |  |  | 0.5 | 2 | 5 |  |  |  |
| Dunstan et al.(49) | TV viewing | <2 | >=2<4 | >=4 |  |  |  | 1 | 3 | 5 |  |  |  |
| Ford et al.(50) | TV viewing | <1 | 1-<2 | 2-<3 | 3-<4 | 4+ |  | 0.5 | 1.5 | 2.5 | 3.5 | 4.5 |  |
| Hu et al. (2001)(52) | TV viewing  (hours/week) | 0-1 | 2-10 | 11-20 | 21-40 | >40 |  | 0.07 | 0.79 | 2.14 | 4.29 | 7.143 |  |
| Hu et al. (2003)(53) | TV viewing  (hours/week) | 0-1 | 2-5 | 6-20 | 21-40 | >40 |  | 0.07 | 0.57 | 1.79 | 4.29 | 7.143 |  |
| Ikehara et al.(54) | TV viewing | <2 | 2 | 3 | 4 | 5 | ≥6 | 1 | 2 | 3 | 4 | 5 | 6.5 |
| Joseph et al.(44) | TV viewing | 0-2 | 2.01-4 | 4.01-6 | >6 |  |  | 1 | 3 | 5 | 7 |  |  |
| Keadle et al.(57) | TV viewing | <1 | 1-2 | 3-4 | 5-6 | >7 |  | 0.5 | 2 | 4 | 6 | 8 |  |
| Kim et al.(45) | TV viewing | <1 | 1-4 | >=5 |  |  |  | 0.5 | 3 | 7 |  |  |  |
| Krishnan et al.(58) | TV viewing | <1 | 1-2 | 3-4 | >5 |  |  | 0.5 | 2 | 4 | 6 |  |  |
| Matthews et al. (2012)(61) | TV viewing | <1 | 1-2 | 3-4 | 5-6 | ≥7 |  | 0.5 | 2 | 4 | 6 | 8 |  |
| Muennig et al.(46) | TV viewing | 0-1 | 2-3 | 4-5 | 6+ |  |  | 1 | 3 | 5 | 7 |  |  |
| Pulsford et al.(66) | TV viewing  (hours/week) | ≥0, <8 | ≥8, <15 | ≥15, <16 | ≥16 |  |  | 0.571 | 1.643 | 2.214 | 2.357 |  |  |
| Smith et al.(69) | TV viewing | <2 | ≥2, <4 | ≥4, <6 | ≥6 |  |  | 1 | 3 | 5 | 7 |  |  |
| Suzuki et al.(70) | TV viewing | <2 | 2-4 | 4+ |  |  |  | 1 | 3 | 5 |  |  |  |
| Warren et al.(47) | TV viewing | <4 | 4-8 | 8-12 | >12 |  |  | 2 | 6 | 10 | 14 |  |  |
| Wijndaele et al.(48) | TV viewing | <2.5 | 2.5-3.6 | >3.6 |  |  |  | 1.25 | 3.05 | 4.15 |  |  |  |

| Appendix Table 4 – additional information about the quality of the 34 studies included in the analysis by outcome | | | | | |  |
| --- | --- | --- | --- | --- | --- | --- |
| Authors | **Inclusion**  **criteria** | **Health related exclusion criteria and/or adjustments** | **Representativeness*** | **Outcome measure** | **Exposure measure** | **Physical activity measurement** |
| *All-cause mortality* | | | | | | |
| Chau et al (2013)(40) | Nord-Trøndelag county residents aged at least 20 years old | No exclusions: adjusted for history of cardio-metabolic disease. | 1 | Norwegian Causes of Death Registry, cause of death using ICD | ‘About how many hours do you sit during an average day? (include work hours and leisure time) ’. Similar to IPAQ which has shown acceptable reliability and validity. Daily time spent watching TV, videos or DVD was assessed with the categories:<1, 1–3, 4–6, >6 h/day. Participants rated their occupational sitting/activity using 4 categories ‘work that mostly involves sitting’; ‘work that requires much walking’; ‘work that requires much walking and lifting’; and ‘heavy physical labour’. This type of categorical occupational sitting/activity measure has been commonly used in other prospective cohort studies. | ‘Do you have at least 30 min of physical activity daily at work or in your leisure time?’ (yes/no). |
| Kim et al (2013)(45) | Drivers' license files in both Hawaii and California. An additional source in Hawaii was the voters' registration file. An additional source of African-Americans in California was the Health Care Financing Administration (HCFA) | Excluded a history of cancer, heart attack or stroke. Excluded those who died within the first year. Adjusted for history of hypertension and/or diabetes | 1 | Death certification linkage and national death index, USA. | Baseline questionnaire (http://www.crch.org/multiethniccohort/ index.htm), sitting activities included: ‘sitting in a car or bus’, ‘sitting at work’, ‘sitting at meals’, ‘sitting watching television’ and ‘other leisure sitting activities e.g. reading’. Each sitting activity was asked in seven categories. | Asked about many different activities and categories into 8 levels of exposure. Light PA estimated by subtracting other activities from 24hours. PA activity (METs/week for moderate activity, vigorous work and strenuous sports. In a validation study, correlation with double label water was "reasonable". |
| Pulsford et al (2015)(66) | The Whitehall II study is a longitudinal study of London based employees of the British Civil Service. At the study’s inception in 1985, all civil servants (aged 35–55) from clerical and office support, middle-ranking executive and senior administrative grades were invited to participate and 73% consented. Sitting asked for the first time in 1997-99 so this represents baseline. | No exclusion, adjusted for physical functioning (SF-36 physical functioning score) to assesses the extent to which participants’ health limits their ability to perform physical activities, ranging in intensity from vigorous (sporting and volitional exercise activities) to light (day-to-day tasks) using the responses ‘a lot’, ‘a little’ and ‘not at all’. Responses were scored, summed and transformed to scale from 0 (limited a lot in performing all types of physical activities) to 100 (able to perform all types of physical activity without limitation). This scale has been demonstrated to have high internal consistency. Also excluded those who died before 2001 and 2003/4 in sensitivity analysis. | 3 | Mortality was established through the national mortality register kept by the National Health Service (NHS) Central Registry. | Participants reported on average how many hours per week they spent: sitting at work including driving or commuting, and sitting at home, e.g., watching TV, sewing, working at a desk, by selecting from eight response categories (none, 1 h, 2–5, 6–10, 11–20, 21–30, 31–40, >40 h). For sitting at home, participants were given an open-text response to specify two sitting behaviours and then select a time category for each. Using the midpoint of these time categories (‘more than 40 h’ was represented as exactly 40 h), five different sitting indicators were computed: (i) work sitting (including commuting); (ii) TV viewing time; (iii) non-TV leisure time sitting; (iv) total leisure time sitting (the sum of ii and iii above); and (v) total sitting time (sum of i–iii above). Although there is no objective criterion measure of context-specific sitting, the questionnaire items used to construct the sitting exposures have demonstrated concurrent validity with past-week recalls (Pearson’s r=0.44), activity diaries (Pearson’s r=0.41)27 and have also been used in a number of previous studies where associations between sitting time and health outcomes have been observed. | Physical activity covariates included daily walking time (min/day) and weekly MVPA (h/week). Physical activity (weekly MVPA) was assessed using a modified version of the Minnesota leisure-time physical activity questionnaire which assesses both occupational and leisure-time activities, and which has been validated previously. Twenty items (including five open-text responses) assessed time spent engaged in walking, sports and games, gardening, housework and do-it-yourself building/maintenance projects, in hours over the previous 4-week period. Each activity was subsequently assigned an energy expenditure value in METs (where 1 MET is equal to energy expenditure at rest) using a compendium of activity energy expenditures. Moderate intensity activities were those eliciting an energy expenditure of 3–5.9 METs and vigorous intensity activities ≥6 METs. Additionally daily walking time (mins/day) was included as a separate covariate. |
| Ensrud et al (2014)(42) | From 2000 to 2002, 5,994 men aged 65 and older were recruited from population-based listings in six regions of the United States for the baseline visit of MrOS, surviving participants were invited to participate in a third study visit. | Excluded men with a history of bilateral hip replacement and men unable to walk without the assistance of another person. Adjusted for comorbidity burden, depressive symptoms, cogitative function, number of instrumental activities of daily living impairments. | 2 | Patients contacted and deaths confirmed with certificates, cause assessed using ICD. | Data were sampled in 1-minute epochs over 24-hour periods from a biaxial accelerometer. These data were used in proprietary algorithms (Interview Professional 5.1 software; BodyMedia, Inc.) along with participant characteristics (age, height, weight, handedness, and smoking status) to estimate time (minutes/24 hours) spent sleeping and time (minutes/24 hours) awake spent in sedentary behaviour (metabolic equivalent (MET) level ≤1.50). A validation study comparing the activity monitor with the criterion method of doubly labelled water showed excellent levels of agreement for total energy expenditure in older adults. Participants were required to have at least five 24- hour periods of data and wear the activity monitor at least 90% of the time to be considered as having valid data; this time period was selected to ensure that the activity monitor recorded time spent in daily activity that was representative of the participant’s usual life. All activity level measures used in the analyses reflected mean daily experience to obtain a representative characterization of usual activity pattern and were averaged over all days to limit variability in the measures. | Physical Activity Scale for the Elderly (PASE). |
| Fox et al (2015)(51) | Recruited from 12 GP surgeries and living in suburban and urban sectors of a large city in south west England, stratified by low, medium or high Index of Multiple Deprivation (IMD) and low or high access to local amenities. Aged 70+ | Screening by a GP for (i) recent bereavement, (ii) terminal illness, (iii) debilitating mental illness, (iv) inability to complete a questionnaire and (v) any other illness preventing participation. Also adjusted for self reported chronic illness and lower limb function. | 2 | QOF, patient records | Physical activity was assessed by 7-day accelerometry using Actigraph GT1Ms programmed to record in 10-s epochs, to produce both count and pedometer data. Data were reduced using MAH/ UFFE Analyser v. 1.9.0.3 (MRC Epidemiology Unit) set to ignore runs of 100 or more zeros, representing time when the monitor was not worn. Inclusion required at least 10 h of monitoring on at least 5 days. Daily means for registered wear time per day, total minutes of activity MVPA (>1,951 CPM), number of steps (STEPS) and minutes of sedentary time (0–99 CPM) were calculated. | none |
| Inoue et al (2008)(55) | All registered Japanese inhabitants in 11 areas age 40-59 (or 69 ) | Excluded those with cancer, stroke, MI due to potential to reduce PA. Adjusted for history of diabetes (Y/N) | 2 | Residence status, including survival was confirmed through the residential registry. Cause of death from death certificate. | Average time spent per day in sedentary activity (3 categories). | Categories of time spent per day in three types of physical activity (heavy physical/strenuous exercise, standing/walking, and leisure-time sports or physical exercise. |
| Katzmarzyk et al (2009)(56) | Representative sample of Canada population excluding some groups e.g. armed forces and institutionalised persons | Physical Activity Readiness Questionnaire (PAR-Q) was included as a covariate (pass/fail/missing). | 1 | Canadian mortality database linkage. | Baseline questionnaire at household visit. The amount of time participants spent sitting during work, school, and housework during the course of most days of the week as either 1) almost none of the time, 2) approximately one fourth of the time, 3) approximately half of the time, 4) approximately three fourths of the time, 5) almost all of the time. | Questions on participation in 20 leisure time activities were used to calculate METh/week continuous variable (logged) and Physical Activity Readiness Questionnaire (PAR-Q) was included as a covariate (pass/fail/missing). The PAR-Q asks several questions regarding heart trouble, chest pain, high blood pressure, dizzy spells, joint problems, and other problems that may prevent participants from participating in physical activities. A positive response to any question results in a failure of the PAR-Q |
| Matthews et al (2014)(60) | From 2002 to 2009, nearly 85,000 adults were enrolled in the cohort, most of whom (86%) enrolled at 1 of 71 participating community health centers that provide basic health services mainly to low-income and uninsured persons. Centres were in urban and rural areas in 12 states in south eastern United States (Florida, Alabama, Mississippi, Louisiana, Arkansas, Tennessee, Georgia, South Carolina, North Carolina, Virginia, West Virginia, and Kentucky). An additional 14% of the cohort enrolled from 2004 to 2006 by responding to a mailed questionnaire sent to randomly selected residents of the same 12 states. The study was designed so that approximately two thirds of participants were black. Participants eligible for enrollment in the overall cohort were 40–79 years of age, spoke English, and had not been treated for cancer in the 12 months before enrollment. | Excluded persons who reported heart disease (previous heart attack or bypass surgery; n = 5,535), cancers other than non-melanoma skin cancer (n = 5,099), stroke (n = 5,150), Parkinson’s disease (n = 130), lupus (n = 674), and multiple sclerosis (n = 255) at enrolment. Adjusted for diabetes (Y/N). | 3 | Social Security Administration and the National Death Index through December 31, 2011. Information on specific causes of death was ascertained via linkage to the National Death Index | Questions about sedentary behaviours asked about the amount of time per day typically spent sitting in a car or bus; sitting at work; sitting to view television or movies; sitting using a computer at home (e.g., checking email, using the Internet, playing games); and doing other activities that involve sitting (e.g., eating meals, talking on the phone, reading, playing cards, or sewing). For all questions, participants provided open-ended duration responses (hours and minutes). We evaluated the reliability and validity of the SCCS physical activity questionnaire in 118 randomly selected SCCS participants and found the validity of the instrument to be comparable for blacks and whites | Time typically spent performing light, moderate, and strenuous (vigorous) activities at home and at work, as well as time spent moderately and vigorously exercising/participating in sports. Time spent doing work and home activities was assessed separately for week and weekend days, and exercise and sports participation was assessed for a typical week. Overall PA level was estimated as the sum of home and work activity and exercise and sports participation. In addition to leisure-time activity and activities done at home and work, time spent walking slowly (moving around, walking at work, walking a dog, doing light exercise) and walking quickly (to go places, for exercise, climbing stairs) was also assessed and evaluated separately. Duration reports of active behaviours were converted to estimates of PA energy expenditure METh/day using common MET values for the specific activities assessed. Reliability and validity of the Southern Community Cohort Study PA questionnaire was evaluated in 118 randomly selected SCCS participants and found the validity of the instrument to be comparable for blacks and whites |
| Matthews et al (2015)(62) | 3.5 million questionnaires to current members of the AARP (formally the American Association of Retired Persons), aged 50-71 years, and who resided in one of six US states or in two metropolitan areas who replied to a questionnaire. | Those with major diseases including cancer other than BCC, MI, coronary bypass, stroke, emphysema, renal dis, degenerative neurological condition. Adjusted for self reported overall health | 3 | Vital status was determined through linkage with the Social Security Administration Death Master File, and the National Death Index | “During a typical 24-hour period over the past 12 months, how much time did you spend watching television or videos? (None, 1, 1–2, 3–4, 5–6, 7–8, or 9+ h/d).” To assess overall sitting, participants were asked, “During a typical 24-hour period over the past 12 months, how much time did you spend sitting? (3, 3–4, 5–6, 7–8, or 9+ h/d).” | The physical activity questionnaire asked how much time per week was spent in 16 activities during the past 12 months. Activities were classified as exercise and sports (8 questions) and as non-exercise activity (8 questions), including household chores (5 questions), lawn and garden (2 questions), and daily walking activities (1 question). Activity duration (hrs/d) was calculated as the sum of all exercise, non-exercise, and all activities (i.e., Overall physical activity), this was used as 5 category variable in the analysis. The exercise items have been validated against physical activity diaries, r=0.62 and 0.65 (3, 34). |
| Pavey et al (2015)(63) | Women were randomly selected from the Medicare database, which covers all citizens and permanent residents of Australia. This analysis focused on the 1921-1926 cohort of the ALSW and were therefore women aged 70-75 at baseline (in 1996). | Too frail to fill in questionnaire and withdrawals. | 2 | Australian National Death Index | How many hours EACH DAY do you typically spend sitting down while doing things like visiting friends, driving, reading, watching television, or working at a desk or computer on (a) a usual week-day and (b) a usual weekend-day”. Similar to that used in IPAQ which has been shown to have good reliability and moderate criterion validity against accelerometers (<100 counts/min).Sitting-time data were cleaned using protocols developed by van Uffelen et al. | PA scores were calculated from questions about time spent in walking, moderate and vigorous activity. (Score=walking+moderate min/ week×3 MET)+(vigorous min/week×6 MET), with scores categorised as: not meeting (<450 MET×min/week) or meeting PA guidelines (≥450 MET×min/week). The PA measure has been shown to have acceptable measurement characteristics. |
| Petersen et al (2014)(64) | DANHES was conducted in 13 municipalities, in each all citizens >18 were invited to participate (response rate 14% 76,484/538,163) | Pre-existing CHD baseline, self-report or using patient register. Also excluded those who rated their general health as very poor (lowest of 5 categories at baseline). | 1 | Danish Civil Registration System (deaths). Danish National Patient Register using ICD. | Long IPAQ Danish. "During the last 7 days how much time did you usually spend sitting during work and leisure time on a) a weekday? b) a weekend day? How much time was spent travelling by car? The sum was converted into hours/day. | LTPA dichotomised into active and inactive with modified version of Saltin and Grimby instrument. Additionally, IPAQ was used to measure LTPA and total PA, total time spent in each domain was used to calculate MET values (31 items in 4 domains: work, transport, house work, gardening and leisure time). |
| Schmid et al (2015)(67) | This study used data from the 2003–2004 NHANES survey, a nationally representative of the US civilian non-institutionalise population. Survey has a stratified, multistage design. | Adjusted for history of diabetes, CVD (CHD, congestive heart failure, stroke), cancer. Sensitivity analysis for those who died in first year of follow up and those with history of chronic diseases and mobility limitations | 1 | Linkage to national death index. | Physical activity was assessed using the uniaxial ActiGraph AM-7164 accelerometer (ActiGraph, Ft. Walton Beach, FL). for up to seven consecutive days. Non-wear time was defined as an interval of at least 60 consecutive minutes of zero counts, allowing for intervals of 1–2 minutes of relatively low counts (i.e., 1–100 counts) [24–27]. To define sedentary time and moderate to vigorous physical activity, we used cut-points from published calibration studies. Specifically, sedentary time was defined as less than 100 counts per minute. | Objective MVPA - defined at 2020 cpm which represents the weighted average cut point for moderate to vigorous physical activity from published calibration studies |
| Seguin et al (2014)(68) | Postmenopausal women aged between 50 and 79 years at clinical centres across the US, through several recruitment activities (e.g., mass mailings, community presentations) and included 93,676 racially and ethnically diverse postmenopausal women aged 50–79 years at baseline. | Adjusted for physical function assessed using self report 10 item Rand-36 | 2 | Mailed questionnaire confirmed by national death index | During a usual day and night, about how many hours do you spend sitting? Be sure to include the time you spend sitting at work, sitting at the table eating, driving or riding in a car or bus, and sitting up watching TV or talking. During a usual day and night, about how many hours do you spend sleeping or lying down with your feet up? Be sure to include the time you spend sleeping or trying to sleep at night, resting or napping, and lying down watching TV. Sleeping time was then subtracted for total sitting time. Demonstrated test-re-test reliability in a subsample. | Respondents were asked to classify the duration, frequency, and intensity of walking and other recreational activities. MVPA was measured using the WHI physical activity questionnaire, which has acceptable validity and reliability. |
| van der Ploeg et al (2012)(71) | Individual aged over 45 years sampled from the general population of NSW Australia, Medicare database which includes all citizens and permanent residents. | Adjustments for self-rated health and receiving help with daily tasks for long term illness or disability. | 2 | New South Wales Registry of Births, Deaths, and Marriages data linkage | About how many hours in each 24-hour day do you usually spend sitting?” similar to IPAQ which has shown acceptable reliability and validity | Total physical activity was assessed with the Active Australia Survey, which measures walking and other moderate- and vigorous-intensity physical activity and has acceptable reliability and validity (4 categories used) |
| Basterra-Gortari et al (2014)(39) | Permanently open recruitment, allowing continuous growth for an ever-increasing sample. Graduate status is necessary, recruited through collaborations with alumni and professional associations throughout the country. 20% of invitees agreed to participate | Excluded those with: CVD, diabetes of cancer at baseline. | 3 | Next of kin notification and National Death Index. | Baseline questionnaire includes time watching TV, using a computer and driving in 12 categories from never to >9hours/day. Measured separately for weekdays and weekends. Validated against objective (accelerometer) measurement. | Total METh/week (Quartiles) based on baseline 17-item questionnaire. Time spent in each activity multiplied by its energy expenditure in METs, then summed. Validated using tri axial accelerometer in a sub-sample. |
| Dunstan et al (2010)(49) | All eligible adults were recruited within 42 randomly selected urban and nonurban areas based on Census Collector Districts, 6 in each of the Australian states and in the Northern Territory of Australia. | Excluded those with history of CVD(CHD or stroke) and those who were pregnant. Adjusted for hypertension, plasma cholesterol, serum triglycerides and glucose tolerance. | 1 | National Death Index using ICD. | Total time spent watching television or videos in the previous 7 days. Validated survey and reliable measure. Three categories used. | Exercise time was measured by the Active Australia questionnaire, which asks respondents about their participation in predominantly leisure-time exercise. "This measure has been shown to provide a reliable and valid estimate of exercise among adults". |
| Keadle et al (2015)(57) | 3.5 million questionnaires to current members of the AARP (formally the American Association of Retired Persons), aged 50-71 years, and who resided in one of six US states or in two metropolitan areas who replied to a questionnaire. | Excluded if had a history of cancer, heart disease, stroke, or emphysema. Additionally adjusted for health status. | 3 | Vital status ascertainment was performed by annual linkage of the cohort to the Social Security Administration Death Master File. Verification of vital status and cause of death were obtained by searches of the National Death Index (NDI) Plus and was available for >95% of the cohort. | During a typical 24-hour period over the past 12 months, how much time did you spend watching television or videos?' | During a typical month in the last 12 months how often did you participant in physical activity at work or at home, including exercise, sports, and activities such as carrying heavy loads? (never or rarely, 1, 1–3, 4–7, >7 h/wk) |
| Matthews et al (2012)(61) | AARP (over 50s membership group) members aged 50–71 y living in California, Florida, Louisiana, New Jersey, North Carolina, Pennsylvania, Atlanta, or Detroit responded to a questionnaire | Excluded those with colon or breast cancer, prior heart disease, cancers other than BCC, stroke, emphysema, rated their health state poor, those who were not filling in their own questionnaire. | 3 | Vital status was determined through linkage with the Social Security Administration Death Master File, and determinations of vital status and causes of death were made by using the National Death Index | “During a typical 24-hour period over the past 12 months, how much time did you spend watching television or videos? (None, 1, 1–2, 3–4, 5–6, 7–8, or 9+ h/d).” To assess overall sitting, participants were asked, “During a typical 24-hour period over the past 12 months, how much time did you spend sitting? (3, 3–4, 5–6, 7–8, or 9+ h/d).”Not evaluated but questions similar to those that have been noted to have acceptable validity. | Leisure time MVPA levels was assessed by asking the average time spent each week in activities of at least moderate intensity in the past 10 years (4 categories). Not evaluated but questions similar to those that have been noted to have acceptable validity. |
| Muennig et al (2013)(46) | A representative sample of the non-institutionalized, U.S., adult population. | Only subject who reported being healthy when surveyed were included. | 1 | National death index linkage. | “On the average day, about how many hours do you personally watch television?” with answers between 0 and 24 hours being valid. | none |
| Suzuki et al (2007)(70) | Those who attended screening and who replied to questionnaire |  | 3 | Cancer registration system | Hours of TV watching/day | Sports participation(h/week), and time of walking (h/week) |
| Wijndaele et al (2011)(48) | Between 1993 and 1997, a cohort of 25 633 residents of Norfolk (UK), in the age range of 45–79 years and recruited via participating general practitioners, agreed to participate. | Self reported history of stroke, MI, other vascular disease and or cancer were excluded. Adjustment for history of diabetes, family history of CVD and family history of cancer. | 2 | Office of National Statistics and death certificates. | EPAQ2 - Time spent watching television and video (hours/week) was calculated based on responses to four questions about watching before and after six pm at week- and weekend-days. The EPAQ2 scored high for repeatability, both in terms of television viewing time and PAEE. It is valid for ranking individuals, as shown by comparison against minute-by-minute heart rate monitoring and maximal aerobic capacity (VO2max). In the current study, television viewing time was expressed in hours/day. | Participants completed the EPAQ2, providing information on their physical (in)activity in a disaggregated way (in and around the home, to work, at work and during leisure time), using the past year as a reference frame. Total PAEE (METh/week) was calculated by summing energy expenditure of the different (mutually exclusive) domains. Repeatability has been shown to be high and validity in ranking individuals has been demonstrated. |
| *CVD mortality* | | | | | | |
| Kim et al (2013)(45) | Drivers' license files in both Hawaii and California. An additional source in Hawaii was the voters' registration file. An additional source of African-Americans in California was the Health Care Financing Administration (HCFA) | Excluded a history of cancer, heart attack or stroke. Excluded those who died within the first year. Adjusted for history of hypertension and/or diabetes | 1 | Death certification linkage and national death index, USA. | Baseline questionnaire (http://www.crch.org/multiethniccohort/ index.htm), sitting activities included: ‘sitting in a car or bus’, ‘sitting at work’, ‘sitting at meals’, ‘sitting watching television’ and ‘other leisure sitting activities e.g. reading’. Each sitting activity was asked in seven categories. | Asked about many different activities and categories into 8 levels of exposure. Light PA estimated by subtracting other activities from 24hours. PA activity (METs/week for moderate activity, vigorous work and strenuous sports. In a validation study, correlation with double label water was "reasonable". |
| Ensrud et al (2014)(42) | From 2000 to 2002, 5,994 men aged 65 and older were recruited from population-based listings in six regions of the United States for the baseline visit of MrOS, surviving participants were invited to participate in a third study visit. | Excluded men with a history of bilateral hip replacement and men unable to walk without the assistance of another person. Adjusted for comorbidity burden, depressive symptoms, cogitative function, number of instrumental activities of daily living impairments. | 2 | Patients contacted and deaths confirmed with certificates, cause assessed using ICD. | Data were sampled in 1-minute epochs over 24-hour periods from a biaxial accelerometer. These data were used in proprietary algorithms (Interview Professional 5.1 software; BodyMedia, Inc.) along with participant characteristics (age, height, weight, handedness, and smoking status) to estimate time (minutes/24 hours) spent sleeping and time (minutes/24 hours) awake spent in sedentary behaviour (metabolic equivalent (MET) level ≤1.50). A validation study comparing the activity monitor with the criterion method of doubly labelled water showed excellent levels of agreement for total energy expenditure in older adults. Participants were required to have at least five 24- hour periods of data and wear the activity monitor at least 90% of the time to be considered as having valid data; this time period was selected to ensure that the activity monitor recorded time spent in daily activity that was representative of the participant’s usual life. All activity level measures used in the analyses reflected mean daily experience to obtain a representative characterization of usual activity pattern and were averaged over all days to limit variability in the measures. | Physical Activity Scale for the Elderly (PASE). |
| Katzmarzyk et al (2009)(56) | Representative sample of Canada population excluding some groups e.g. armed forces and institutionalised persons | Physical Activity Readiness Questionnaire (PAR-Q) was included as a covariate (pass/fail/missing). | 1 | Canadian mortality database linkage. | Baseline questionnaire at household visit. The amount of time participants spent sitting during work, school, and housework during the course of most days of the week as either 1) almost none of the time, 2) approximately one fourth of the time, 3) approximately half of the time, 4) approximately three fourths of the time, 5) almost all of the time. | Questions on participation in 20 leisure time activities were used to calculate METh/week continuous variable (logged) and Physical Activity Readiness Questionnaire (PAR-Q) was included as a covariate (pass/fail/missing). The PAR-Q asks several questions regarding heart trouble, chest pain, high blood pressure, dizzy spells, joint problems, and other problems that may prevent participants from participating in physical activities. A positive response to any question results in a failure of the PAR-Q |
| Matthews et al (2014)(60) | From 2002 to 2009, nearly 85,000 adults were enrolled in the cohort, most of whom (86%) enrolled at 1 of 71 participating community health centers that provide basic health services mainly to low-income and uninsured persons. Centres were in urban and rural areas in 12 states in south eastern United States (Florida, Alabama, Mississippi, Louisiana, Arkansas, Tennessee, Georgia, South Carolina, North Carolina, Virginia, West Virginia, and Kentucky). An additional 14% of the cohort enrolled from 2004 to 2006 by responding to a mailed questionnaire sent to randomly selected residents of the same 12 states. The study was designed so that approximately two thirds of participants were black. Participants eligible for enrollment in the overall cohort were 40–79 years of age, spoke English, and had not been treated for cancer in the 12 months before enrollment. | Excluded persons who reported heart disease (previous heart attack or bypass surgery; n = 5,535), cancers other than non-melanoma skin cancer (n = 5,099), stroke (n = 5,150), Parkinson’s disease (n = 130), lupus (n = 674), and multiple sclerosis (n = 255) at enrolment. Adjusted for diabetes (Y/N). | 3 | Social Security Administration and the National Death Index through December 31, 2011. Information on specific causes of death was ascertained via linkage to the National Death Index | Questions about sedentary behaviours asked about the amount of time per day typically spent sitting in a car or bus; sitting at work; sitting to view television or movies; sitting using a computer at home (e.g., checking email, using the Internet, playing games); and doing other activities that involve sitting (e.g., eating meals, talking on the phone, reading, playing cards, or sewing). For all questions, participants provided open-ended duration responses (hours and minutes). We evaluated the reliability and validity of the SCCS physical activity questionnaire in 118 randomly selected SCCS participants and found the validity of the instrument to be comparable for blacks and whites | Time typically spent performing light, moderate, and strenuous (vigorous) activities at home and at work, as well as time spent moderately and vigorously exercising/participating in sports. Time spent doing work and home activities was assessed separately for week and weekend days, and exercise and sports participation was assessed for a typical week. Overall PA level was estimated as the sum of home and work activity and exercise and sports participation. In addition to leisure-time activity and activities done at home and work, time spent walking slowly (moving around, walking at work, walking a dog, doing light exercise) and walking quickly (to go places, for exercise, climbing stairs) was also assessed and evaluated separately. Duration reports of active behaviours were converted to estimates of PA energy expenditure METh/day using common MET values for the specific activities assessed. Reliability and validity of the Southern Community Cohort Study PA questionnaire was evaluated in 118 randomly selected SCCS participants and found the validity of the instrument to be comparable for blacks and whites |
| Matthews et al (2015)(62) | 3.5 million questionnaires to current members of the AARP (formally the American Association of Retired Persons), aged 50-71 years, and who resided in one of six US states or in two metropolitan areas who replied to a questionnaire. | Those with major diseases including cancer other than BCC, MI, coronary bypass, stroke, emphysema, renal dis, degenerative neurological condition. Adjusted for self reported overall health | 3 | Vital status was determined through linkage with the Social Security Administration Death Master File, and the National Death Index | “During a typical 24-hour period over the past 12 months, how much time did you spend watching television or videos? (None, 1, 1–2, 3–4, 5–6, 7–8, or 9+ h/d).” To assess overall sitting, participants were asked, “During a typical 24-hour period over the past 12 months, how much time did you spend sitting? (3, 3–4, 5–6, 7–8, or 9+ h/d).” | The physical activity questionnaire asked how much time per week was spent in 16 activities during the past 12 months. Activities were classified as exercise and sports (8 questions) and as non-exercise activity (8 questions), including household chores (5 questions), lawn and garden (2 questions), and daily walking activities (1 question). Activity duration (hrs/d) was calculated as the sum of all exercise, non-exercise, and all activities (i.e., Overall physical activity), this was used as 5 category variable in the analysis. The exercise items have been validated against physical activity diaries, r=0.62 and 0.65 (3, 34). |
| Seguin et al (2014)(68) | Postmenopausal women aged between 50 and 79 years at clinical centres across the US, through several recruitment activities (e.g., mass mailings, community presentations) and included 93,676 racially and ethnically diverse postmenopausal women aged 50–79 years at baseline. | Adjusted for physical function assessed using self report 10 item Rand-36 | 2 | Mailed questionnaire confirmed by national death index | During a usual day and night, about how many hours do you spend sitting? Be sure to include the time you spend sitting at work, sitting at the table eating, driving or riding in a car or bus, and sitting up watching TV or talking. During a usual day and night, about how many hours do you spend sleeping or lying down with your feet up? Be sure to include the time you spend sleeping or trying to sleep at night, resting or napping, and lying down watching TV. Sleeping time was then subtracted for total sitting time. Demonstrated test-re-test reliability in a subsample. | Respondents were asked to classify the duration, frequency, and intensity of walking and other recreational activities. MVPA was measured using the WHI physical activity questionnaire, which has acceptable validity and reliability. |
| Dunstan et al (2010)(49) | All eligible adults were recruited within 42 randomly selected urban and nonurban areas based on Census Collector Districts, 6 in each of the Australian states and in the Northern Territory of Australia. | Excluded those with history of CVD(CHD or stroke) and those who were pregnant. Adjusted for hypertension, plasma cholesterol, serum triglycerides and glucose tolerance. | 1 | National Death Index using ICD. | Total time spent watching television or videos in the previous 7 days. Validated survey and reliable measure. Three categories used. | Exercise time was measured by the Active Australia questionnaire, which asks respondents about their participation in predominantly leisure-time exercise. "This measure has been shown to provide a reliable and valid estimate of exercise among adults". |
| Ikehara et al (2015)(54) | All non-institutionalized residents from 45 area throughout Japan aged 40–79 years in communities were invited to participate in the survey. The participation rate was between 86% and 91%. In most regions, informed consent was obtained individually and directly from members of the cohort, while in several areas, informed consent was obtained at the community level after the purpose of the study and confidentiality of the data had been explained to community leaders and mayors. Most of the participants were people who had received a municipal health checkup. | Of the 110,585 cohort participants, 5,850 subjects (2,574 men; 3,276 women) who reported a history of CVD and cancer were excluded. In addition adjustment was made for perceived stress and depressive symptoms. | 1 | Systematic review of death certificates in the participating communities. Coded using ICD-10. Subjects who moved out of survey areas were censored. | Baseline questionnaire: ‘On average how many hours do you watch TV?’ The participants reported their time spent viewing TV a day: ‘approximately h/day.’ TV viewing time was then classified into 6 categories: <2 h, 2 h, 3 h, 4 h, 5 h and ≥6 h/day. | Baseline questionnaire question about hours of sport per week. Also a separate variable of amount of walking. |
| Matthews et al (2012)(61) | AARP (over 50s membership group) members aged 50–71 y living in California, Florida, Louisiana, New Jersey, North Carolina, Pennsylvania, Atlanta, or Detroit responded to a questionnaire | Excluded those with colon or breast cancer, prior heart disease, cancers other than BCC, stroke, emphysema, rated their health state poor, those who were not filling in their own questionnaire. | 3 | Vital status was determined through linkage with the Social Security Administration Death Master File, and determinations of vital status and causes of death were made by using the National Death Index | “During a typical 24-hour period over the past 12 months, how much time did you spend watching television or videos? (None, 1, 1–2, 3–4, 5–6, 7–8, or 9+ h/d).” To assess overall sitting, participants were asked, “During a typical 24-hour period over the past 12 months, how much time did you spend sitting? (3, 3–4, 5–6, 7–8, or 9+ h/d).”Not evaluated but questions similar to those that have been noted to have acceptable validity. | Leisure time MVPA levels was assessed by asking the average time spent each week in activities of at least moderate intensity in the past 10 years (4 categories). Not evaluated but questions similar to those that have been noted to have acceptable validity. |
| Warren et al (2010)(47) | Healthy male adults. Participants were self-referred or employer referred to the clinic for various services such as preventive medical examinations and health, nutrition, and exercise counselling. | Excluded with history of MI, stroke or cancer. Adjustment for family history of CVD, hypertension, diabetes and hypercholesterolemia. | 3 | National death index augmented with death certificates. | Average time h/week viewing TV at baseline. | Physically inactive or active was assessed by questions asking the participant to rate their physical activity level (including both leisure and work activities) as compared with others of the same age and sex. Those who answered ‘‘extremely inactive,’’ ‘‘inactive,’’ or ‘‘somewhat inactive’’ were classified as physically inactive, whereas others who reported ‘‘about average,’’ ‘‘somewhat active,’’ ‘‘active,’’ or ‘‘extremely active’’ were classified physically active. |
| Wijndaele et al (2011)(48) | Between 1993 and 1997, a cohort of 25 633 residents of Norfolk (UK), in the age range of 45–79 years and recruited via participating general practitioners, agreed to participate. | Self reported history of stroke, MI, other vascular disease and or cancer were excluded. Adjustment for history of diabetes, family history of CVD and family history of cancer. | 2 | Office of National Statistics and death certificates. | EPAQ2 - Time spent watching television and video (hours/week) was calculated based on responses to four questions about watching before and after six pm at week- and weekend-days. The EPAQ2 scored high for repeatability, both in terms of television viewing time and PAEE. It is valid for ranking individuals, as shown by comparison against minute-by-minute heart rate monitoring and maximal aerobic capacity (VO2max). In the current study, television viewing time was expressed in hours/day. | Participants completed the EPAQ2, providing information on their physical (in)activity in a disaggregated way (in and around the home, to work, at work and during leisure time), using the past year as a reference frame. Total PAEE (METh/week) was calculated by summing energy expenditure of the different (mutually exclusive) domains. Repeatability has been shown to be high and validity in ranking individuals has been demonstrated. |
| *Cancer Mortality* | | | | | | |
| Kim et al (2013)(45) | Drivers' license files in both Hawaii and California. An additional source in Hawaii was the voters' registration file. An additional source of African-Americans in California was the Health Care Financing Administration (HCFA) | Excluded a history of cancer, heart attack or stroke. Excluded those who died within the first year. Adjusted for history of hypertension and/or diabetes | 1 | Death certification linkage and national death index, USA. | Baseline questionnaire (http://www.crch.org/multiethniccohort/ index.htm), sitting activities included: ‘sitting in a car or bus’, ‘sitting at work’, ‘sitting at meals’, ‘sitting watching television’ and ‘other leisure sitting activities e.g. reading’. Each sitting activity was asked in seven categories. | Asked about many different activities and categories into 8 levels of exposure. Light PA estimated by subtracting other activities from 24hours. PA activity (METs/week for moderate activity, vigorous work and strenuous sports. In a validation study, correlation with double label water was "reasonable". |
| Ensrud et al (2014)(42) | From 2000 to 2002, 5,994 men aged 65 and older were recruited from population-based listings in six regions of the United States for the baseline visit of MrOS, surviving participants were invited to participate in a third study visit. | Excluded men with a history of bilateral hip replacement and men unable to walk without the assistance of another person. Adjusted for comorbidity burden, depressive symptoms, cogitative function, number of instrumental activities of daily living impairments. | 2 | Patients contacted and deaths confirmed with certificates, cause assessed using ICD. | Data were sampled in 1-minute epochs over 24-hour periods from a biaxial accelerometer. These data were used in proprietary algorithms (Interview Professional 5.1 software; BodyMedia, Inc.) along with participant characteristics (age, height, weight, handedness, and smoking status) to estimate time (minutes/24 hours) spent sleeping and time (minutes/24 hours) awake spent in sedentary behaviour (metabolic equivalent (MET) level ≤1.50). A validation study comparing the activity monitor with the criterion method of doubly labelled water showed excellent levels of agreement for total energy expenditure in older adults. Participants were required to have at least five 24- hour periods of data and wear the activity monitor at least 90% of the time to be considered as having valid data; this time period was selected to ensure that the activity monitor recorded time spent in daily activity that was representative of the participant’s usual life. All activity level measures used in the analyses reflected mean daily experience to obtain a representative characterization of usual activity pattern and were averaged over all days to limit variability in the measures. | Physical Activity Scale for the Elderly (PASE). |
| Katzmarzyk et al (2009)(56) | Representative sample of Canada population excluding some groups e.g. armed forces and institutionalised persons | Physical Activity Readiness Questionnaire (PAR-Q) was included as a covariate (pass/fail/missing). | 1 | Canadian mortality database linkage. | Baseline questionnaire at household visit. The amount of time participants spent sitting during work, school, and housework during the course of most days of the week as either 1) almost none of the time, 2) approximately one fourth of the time, 3) approximately half of the time, 4) approximately three fourths of the time, 5) almost all of the time. | Questions on participation in 20 leisure time activities were used to calculate METh/week continuous variable (logged) and Physical Activity Readiness Questionnaire (PAR-Q) was included as a covariate (pass/fail/missing). The PAR-Q asks several questions regarding heart trouble, chest pain, high blood pressure, dizzy spells, joint problems, and other problems that may prevent participants from participating in physical activities. A positive response to any question results in a failure of the PAR-Q |
| Matthews et al (2014)(60) | From 2002 to 2009, nearly 85,000 adults were enrolled in the cohort, most of whom (86%) enrolled at 1 of 71 participating community health centers that provide basic health services mainly to low-income and uninsured persons. Centres were in urban and rural areas in 12 states in south eastern United States (Florida, Alabama, Mississippi, Louisiana, Arkansas, Tennessee, Georgia, South Carolina, North Carolina, Virginia, West Virginia, and Kentucky). An additional 14% of the cohort enrolled from 2004 to 2006 by responding to a mailed questionnaire sent to randomly selected residents of the same 12 states. The study was designed so that approximately two thirds of participants were black. Participants eligible for enrollment in the overall cohort were 40–79 years of age, spoke English, and had not been treated for cancer in the 12 months before enrollment. | Excluded persons who reported heart disease (previous heart attack or bypass surgery; n = 5,535), cancers other than non-melanoma skin cancer (n = 5,099), stroke (n = 5,150), Parkinson’s disease (n = 130), lupus (n = 674), and multiple sclerosis (n = 255) at enrolment. Adjusted for diabetes (Y/N). | 3 | Social Security Administration and the National Death Index through December 31, 2011. Information on specific causes of death was ascertained via linkage to the National Death Index | Questions about sedentary behaviours asked about the amount of time per day typically spent sitting in a car or bus; sitting at work; sitting to view television or movies; sitting using a computer at home (e.g., checking email, using the Internet, playing games); and doing other activities that involve sitting (e.g., eating meals, talking on the phone, reading, playing cards, or sewing). For all questions, participants provided open-ended duration responses (hours and minutes). We evaluated the reliability and validity of the SCCS physical activity questionnaire in 118 randomly selected SCCS participants and found the validity of the instrument to be comparable for blacks and whites | Time typically spent performing light, moderate, and strenuous (vigorous) activities at home and at work, as well as time spent moderately and vigorously exercising/participating in sports. Time spent doing work and home activities was assessed separately for week and weekend days, and exercise and sports participation was assessed for a typical week. Overall PA level was estimated as the sum of home and work activity and exercise and sports participation. In addition to leisure-time activity and activities done at home and work, time spent walking slowly (moving around, walking at work, walking a dog, doing light exercise) and walking quickly (to go places, for exercise, climbing stairs) was also assessed and evaluated separately. Duration reports of active behaviours were converted to estimates of PA energy expenditure METh/day using common MET values for the specific activities assessed. Reliability and validity of the Southern Community Cohort Study PA questionnaire was evaluated in 118 randomly selected SCCS participants and found the validity of the instrument to be comparable for blacks and whites |
| Matthews et al (2015)(62) | 3.5 million questionnaires to current members of the AARP (formally the American Association of Retired Persons), aged 50-71 years, and who resided in one of six US states or in two metropolitan areas who replied to a questionnaire. | Those with major diseases including cancer other than BCC, MI, coronary bypass, stroke, emphysema, renal dis, degenerative neurological condition. Adjusted for self-reported overall health | 3 | Vital status was determined through linkage with the Social Security Administration Death Master File, and the National Death Index | “During a typical 24-hour period over the past 12 months, how much time did you spend watching television or videos? (None, 1, 1–2, 3–4, 5–6, 7–8, or 9+ h/d).” To assess overall sitting, participants were asked, “During a typical 24-hour period over the past 12 months, how much time did you spend sitting? (3, 3–4, 5–6, 7–8, or 9+ h/d).” | The physical activity questionnaire asked how much time per week was spent in 16 activities during the past 12 months. Activities were classified as exercise and sports (8 questions) and as non-exercise activity (8 questions), including household chores (5 questions), lawn and garden (2 questions), and daily walking activities (1 question). Activity duration (hrs/d) was calculated as the sum of all exercise, non-exercise, and all activities (i.e., Overall physical activity), this was used as 5 category variable in the analysis. The exercise items have been validated against physical activity diaries, r=0.62 and 0.65 (3, 34). |
| Seguin et al (2014)(68) | Postmenopausal women aged between 50 and 79 years at clinical centres across the US, through several recruitment activities (e.g., mass mailings, community presentations) and included 93,676 racially and ethnically diverse postmenopausal women aged 50–79 years at baseline. | Adjusted for physical function assessed using self report 10 item Rand-36 | 2 | Mailed questionnaire confirmed by national death index | During a usual day and night, about how many hours do you spend sitting? Be sure to include the time you spend sitting at work, sitting at the table eating, driving or riding in a car or bus, and sitting up watching TV or talking. During a usual day and night, about how many hours do you spend sleeping or lying down with your feet up? Be sure to include the time you spend sleeping or trying to sleep at night, resting or napping, and lying down watching TV. Sleeping time was then subtracted for total sitting time. Demonstrated test-re-test reliability in a subsample. | Respondents were asked to classify the duration, frequency, and intensity of walking and other recreational activities. MVPA was measured using the WHI physical activity questionnaire, which has acceptable validity and reliability. |
| Dunstan et al (2010)(49) | All eligible adults were recruited within 42 randomly selected urban and nonurban areas based on Census Collector Districts, 6 in each of the Australian states and in the Northern Territory of Australia. | Excluded those with history of CVD(CHD or stroke) and those who were pregnant. Adjusted for hypertension, plasma cholesterol, serum triglycerides and glucose tolerance. | 1 | National Death Index using ICD. | Total time spent watching television or videos in the previous 7 days. Validated survey and reliable measure. Three categories used. | Exercise time was measured by the Active Australia questionnaire, which asks respondents about their participation in predominantly leisure-time exercise. "This measure has been shown to provide a reliable and valid estimate of exercise among adults". |
| Keadle et al (2015)(57) | 3.5 million questionnaires to current members of the AARP (formally the American Association of Retired Persons), aged 50-71 years, and who resided in one of six US states or in two metropolitan areas who replied to a questionnaire. | Excluded if had a history of cancer, heart disease, stroke, or emphysema. Additionally adjusted for health status. | 3 | Vital status ascertainment was performed by annual linkage of the cohort to the Social Security Administration Death Master File. Verification of vital status and cause of death were obtained by searches of the National Death Index (NDI) Plus and was available for >95% of the cohort. | During a typical 24-hour period over the past 12 months, how much time did you spend watching television or videos?' | During a typical month in the last 12 months how often did you particpant in physical activity at work or at home, including exercise, sports, and activities such as carrying heavy loads? (never or rarely, 1, 1–3, 4–7, >7 h/wk) |
| Matthews et al (2012)(61) | AARP (over 50s membership group) members aged 50–71 y living in California, Florida, Louisiana, New Jersey, North Carolina, Pennsylvania, Atlanta, or Detroit responded to a questionnaire | Excluded those with colon or breast cancer, prior heart disease, cancers other than BCC, stroke, emphysema, rated their health state poor, those who were not filling in their own questionnaire. | 3 | Vital status was determined through linkage with the Social Security Administration Death Master File, and determinations of vital status and causes of death were made by using the National Death Index | “During a typical 24-hour period over the past 12 months, how much time did you spend watching television or videos? (None, 1, 1–2, 3–4, 5–6, 7–8, or 9+ h/d).” To assess overall sitting, participants were asked, “During a typical 24-hour period over the past 12 months, how much time did you spend sitting? (3, 3–4, 5–6, 7–8, or 9+ h/d).”Not evaluated but questions similar to those that have been noted to have acceptable validity. | Leisure time MVPA levels was assessed by asking the average time spent each week in activities of at least moderate intensity in the past 10 years (4 categories). Not evaluated but questions similar to those that have been noted to have acceptable validity. |
| Suzuki et al (2007)(70) | Those who attended screening and who replied to questionnaire |  | 3 | Cancer registration system | Hours of TV watching/day | Sports participation(h/week), and time of walking (h/week) |
| Wijndaele et al (2011)(48) | Between 1993 and 1997, a cohort of 25 633 residents of Norfolk (UK), in the age range of 45–79 years and recruited via participating general practitioners, agreed to participate. | Self reported history of stroke, MI, other vascular disease and or cancer were excluded. Adjustment for history of diabetes, family history of CVD and family history of cancer. | 2 | Office of National Statistics and death certificates. | EPAQ2 - Time spent watching television and video (hours/week) was calculated based on responses to four questions about watching before and after six pm at week- and weekend-days. The EPAQ2 scored high for repeatability, both in terms of television viewing time and PAEE. It is valid for ranking individuals, as shown by comparison against minute-by-minute heart rate monitoring and maximal aerobic capacity (VO2max). In the current study, television viewing time was expressed in hours/day. | Participants completed the EPAQ2, providing information on their physical (in)activity in a disaggregated way (in and around the home, to work, at work and during leisure time), using the past year as a reference frame. Total PAEE (METh/week) was calculated by summing energy expenditure of the different (mutually exclusive) domains. Repeatability has been shown to be high and validity in ranking individuals has been demonstrated. |
| *Incident Type 2 diabetes* | | | | | | |
| Ding et al (2015)(41) | Participants were randomly sampled from the Medicare Australia (national health insurance) database, which included information on all Australian citizens and permanent residents, and some temporary residents and refugees who were residents of NSW | Participants with diabetes were excluded. Additional adjustment was made for CVD, hypertension, high cholesterol, family history of T2DM. | 1 | Respondents were asked whether they had ever been told by a doctor that they had diabetes and, if so, their age or year of diagnosis. A previous study from the same cohort validated the self-report of diabetes against linked administrative data on hospitalization, medical services, and pharmaceuticals and found that reported diagnosis of diabetes in the 45 and Up study had high sensitivity and specificity | Daily sitting and sleeping time were measured using separate single-item questions about the total number of hours spent sitting/sleeping in the last 24 h. | Physical activity was assessed using the validated Active Australia Survey and the total physical activity volume was calculated as the sum of time spent in walking, moderate-intensity physical activity, and vigorous- intensity physical activity (weighted by a factor of two) in the past week. Total weekly physical activity time was categorized using cut-off points of 150 and 300 min to reflect the current physical activity guidelines for Australia. |
| Gibbs et at (2015)(43) | Aimed to get as representative sample as possible of underlying populations of black and white adults aged 18-30 stratified on a number of variables. Recruited by telephone. The sample for the current report includes participants enrolled in the CARDIA year 20 Fitness substudy and who had >4 days with >10 h of accelerometry data. | Those with baseline diabetes were excluded from this analysis. | 1 | Diabetes was defined as either self-reported use of diabetes medications, HbA1c ≥6.5% (≥47.5 mmol/mol), fasting glucose ≥126 mg/dL, or 2-h glucose ≥200 mg/dL. Although we did not have information on type of diabetes, only n = 9 and n = 11 cases of diabetes were present at the CARDIA exams occurring when subjects were 18–30 and 23–35 years old, suggesting few (≈5% of total cases) might have had type 1 diabetes. | NHANES cut points were used to classify total duration of sedentary behaviour (0–99 cpm). | National Health and Nutrition Examination Survey (NHANES) cut points were used to classify total duration of MVPA (≥2,020 cpm). |
| Manini et al (2014)(59) | Postmenopausal women aged between 50 and 79 years at clinical centres across the US, through several recruitment activities (e.g., mass mailings, community presentations) and included 93,676 racially and ethnically diverse postmenopausal women aged 50–79 years at baseline. | Any medical condition associated with predicted survival of less than 3 years, alcoholism, mental illness, or dementia. | 2 | Incident-treated diabetes was ascertained and defined as the self-report of a new physician diagnosis of diabetes treated with oral pills or insulin. Unfortunately, information about diabetes without treatment for pills or insulin (i.e., with lifestyle intervention) was not ascertained at follow-up evaluations. Self-reported incident treated diabetes was found to be concordant with medication inventory in 72% of women in the WHI OS study, while > 99% of those women not reporting diabetes had no evidence of antidiabetic medications or insulin in their medication inventory. | Amount of daily sitting time was measured by asking, “During a usual day and night, about how many hours do you spend sitting? Be sure to include the time you spend sitting at work, sitting at the table eating, driving or riding in a car or bus, and sitting up watching TV or talking.” Eight categories were available for women to choose: <4, 4–5, 6–7, 8–9, 10–11, 12–13, 14–15, and >16 hours. These categories were collapsed to four (<7, 8–11, 12–15, and 16 or more hours) categories | MVPA was determined by using a detailed questionnaire on the frequency and duration of walking and other types of activity. Walking was assessed by a series of questions about the frequency of walks outside the home for > 10 minutes without stopping. The average duration of each walk and the usual walking pace was recorded. Vigorous exercise was defined as that in which “you work up a sweat and your heart beats fast,” and examples included aerobics, aerobic dancing, jogging, tennis, and swimming laps. Moderate exercise was defined as that which was “not exhausting,” and examples included biking outdoors, using an exercise machine (such as a stationary bicycle or a treadmill), calisthenics, easy swimming, and popular or folk dancing. Total weekly physical activity energy expenditure was calculated as the summed product of frequency, duration, and intensity for reported activities. The duration, in minutes per week performing MVPA, was categorized into three levels: none (0 minutes), 1–149 minutes per week and <150 min/week. |
| Petersen et al (2016)(65) | In 13 municipalities, all citizens aged 18 years or more were invited to participate by letter. No reminders were sent. The study was conducted over a period of 1 month in each municipality. A total of 538 163 persons were invited, and 76 478 persons participated (response rate: 14%). | Previous diabetes, patients with poor self-reported health were excluded from this analysis. Adjustment was made for hypertension and previous CVD. | 1 | Diabetes was defined if one of the following criteria was fulfilled: hospitalisation with a diagnosis of diabetes (ICD 8: 249 or 250, ICD-10: DE10–14, DH 36.0 or DO24 (excluding D=24.4)); registration of chiropody (coded for diabetes) in the National Health Insurance Service Registry; frequent measurements of blood glucose either at least five times within 365 days, or at least two annual measurements of glucose during a 5-year period (registered in the National Health Insurance Service Registry); or prescription of insulin or oral antidiabetic medication at least twice (from the Register of Medicinal Product Statistics) | During the past 7 days, how much time did you usually spend sitting during work and leisure time on: (1) a weekday? and (2) a weekend day?' | In all four domains, work, transport, housework/gardening and leisure time, participants were asked to indicate the amount of time spent on physical activity during the previous 7 days in relation to frequency (days/week), duration (in h and min) and level of intensity (walking, moderate and vigorous). Time spent in MVPAs in leisure time and for transportation was categorised into four categories: <30, 30 to <150, 150 to <300 and 300+ min/week. In addition to MVPA, time spent in walking activities was estimated but this was not used as a covariate in the sedentary analysis. |
| Anjana et al (2015)(38) | Individuals of both genders aged 20 years and above, representative of Chennai. A total of 26,000 adults were recruited from 46 Corporation wards using the systematic sampling technique. | Those with diabetes at baseline were excluded. Adjusted for family history of diabetes. | 1 | History of diabetes during the follow-up period was obtained through self-report and checked against medical records for validity. At the follow-up visit, a venous blood sample was drawn in the fasting state and 2 h after oral administration of 75 g of glucose to ascertain the diabetes status of all individuals who did not report a history of development of diabetes in the interim. Diabetes was diagnosed if the venous plasma glucose 2 h after oral glucose load was 11.1 mmol/l (≥200 mg/dl) and/or the fasting plasma glucose levels were 7.0 mmol/l (≥126 mg/dl) | Questionnaire ascertained TV viewing was represented in quartiles of hours/day | The questions on physical activity were used to assess frequency, intensity and duration of various activities in the work, transport and recreational domains. Physical activity was dichotomously coded as active (moderate or vigorous intensity physical activity achieving at least 600 metabolic equivalent [MET]–minutes per week) or inactive (not meeting the above criteria) |
| Ford et al (2010)(50) | Men aged 40–65 years and women aged 35–65 years in the general public of Potsdam were the target of recruitment and joined the study between 1994 and 1998. | Diabetes at baseline | 2 | Potential cases identified with self-report, confirmed with diagnosis physician. | Question administered at baseline: ‘‘On average, how many h ⁄ day did you watch television during the last 12 months?’’ | Physical activity h/week plus physical strain at work. The physical activity questions, which were developed for the EPIC study, included questions about bicycling, sports activities, and gardening. |
| Hu et al (2001)(52) | Male health professionals (dentists, optometrists, pharmacists, podiatrists, osteopaths and veterinarians, aged 40-75. | Excluded were those with previous diagnosis of CVD, Cancer or diabetes at baseline - CVD and cancer due to their effect on PA levels. | 3 | A case of diabetes was confirmed by questionnaire. If the answer to any of the following was affirmative: 1) 1 or more classic symptom (excessive thirst, polyuria etc) plus fasting glucose >7.8mmol/l, 2) at least 2 elevated plasma glucose conc. 3)treatment with hypoglycaemic meds. | 1988 reported average weekly time spent watching TV inc. video-tapes, the analysis used 5 response categories | Quintiles. Assessed ev 2 yrs using mailed questionnaire asking about: walking, jogging, running, cycling, calisthenics or rowing machine, swimming, squash or racketball and tennis, they were also asked about walking pace. From this information MET/hrs was calculated. The measure has been validated using a subsample of this cohort and elsewhere. |
| Hu et al (2003)(53) | Female registered nurses aged 30-55 residing in 11 US states mailed questionnaires | Excluded were those with previously diagnosed with CVD, cancer, diabetes in 1992 | 3 | A case of diabetes was confirmed by questionnaire. If the answer to any of the following was affirmative: 1) 1 or more classic symptom (excessive thirst, polyuria etc) plus fasting glucose >7.8mmol/l, 2) at least 2 elevated plasma glucose conc. 3)treatment with hypoglycaemic meds. | 1992 questionnaire asked average time spent sitting at home watching TV or vcr, sitting at work or away from home or while driving and other sitting at home e.g. reading, mealtimes, at desk. % response categories used. | Quintiles. Assessed ev 2 yrs using mailed questionnaire asking about: walking, jogging, running, cycling, calisthenics or rowing machine, swimming, squash or racketball and tennis, they were also asked about walking pace. From this information MET/hrs was calculated. The measure has been validated using a subsample of this cohort and elsewhere. |
| Joseph et al (2016)(44) | A multistage, stratified random selection of households at 6 selected regions. Men and women, in equal numbers, who are aged 45–84 years and free of clinical CVD at baseline, including four racial/ethnic groups from six US communities. Approximately 38 percent of the cohort will be White, 28 percent will be African-American, 23 percent will be Hispanic, and 11 percent will be Asian, predominantly of Chinese descent. | Free of CVD at baseline. Additionally adjusted for systolic blood pressure, and current hypertension medication usage. | 1 | Persons newly using hypoglycemic medication (per the medication inventory) or having fasting glucose ≥7 mmol/L (126 mg/dL) at one of four subsequent examinations (the last follow-up visit occurring in 2010–2012) were considered to have incident type 2 diabetes. | TV watching (hours/day) 0-2, 2.01-4, 4.01-6, >6. | The MESA Typical Week Physical Activity Survey (TWPAS), adapted from the Cross-Cultural Activity Participation Study, was designed to identify the time and frequency spent in various PAs during a typical week in the past month.13 The MESA TWPAS had the following summary measures: total minutes/week and total metabolic equivalents of task (MET)-hours/week at three intensity levels (light, moderate, vigorous). The survey also inquired about the typical pace at which participants walked in five categories ranging from very slow to brisk. |
| Krishnan et al (2009)(58) | Mailed to readers of Essence magazine and several professional organisations, and friends and relatives of early responders. Aged 21-69 years. | Excluded those with a history of diabetes, gestational diabetes, reported stroke, MI or cancer. Also excluded those who were pregnant. Adjusted for family history of diabetes. | 3 | Questionnaire asking about new diagnoses. Subsample to validate indicated 96% accuracy. | The number of hours per day spent watching television was also assessed in each questionnaire with 5 possible response categories | Hours/week in vigorous activity (e.g. running, swimming), walking for exercise and walking to and from work in time categories. Pace of walking was asked some measure combining pace and amount of walking. Cumulative average vigorous PA and walking were calculated using as many years data as were available. |
| Smith et al (2014)(69) | Representative sample of population living in households, born on or before Feb 1952, UK | Previous diabetes. Adjustment for depressive symptoms, long standing illness, disability. | 2 | Self reported physician diagnoses diabetes, which has been previous validated in ELSA | How many hours of television do you watch on an ordinary day or evening, that is, Monday to Friday’ and ‘How many hours of television do you normally watch in total over the weekend, that is, Saturday and Sunday’. | Participants were asked how often they took part in vigorous, moderate- and low-intensity physical activity, using prompt cards to help them interpret different physical activity intensities. Response options were: more than once a week, once a week, one to three times a month and hardly ever/never. Physical activity was further categorized into three categories: inactive; moderate activity at least once a week; and vigorous activity at least once a week. The physical activity and television viewing measures have been shown to have excellent convergent validity in grading a plethora of psychosocial, physical and biochemical risk factors. |
| *1: Reasonably representative sample of the general population 2:Taken from the general population but likely to be significantly bias e.g. older people. 3: Sampled from an unrepresentative group e.g. Nurses | | | | | | |

Appendix Figure 3 – Non-linear associations between sedentary behaviours and health outcome without adjustment for physical activity

.

Appendix Figure 4 – Non-linear associations between sedentary behaviours and health outcome with adjustment for physical activity

| Appendix Table 5. Relative risks (95% CI) below and above the exposure level corresponding with a change in strength of association, for all-cause and CVD mortality | | | |
| --- | --- | --- | --- |
|  |  | **Below Threshold** | **Above Threshold** |
| **All-cause mortality** | **Total Sedentary behaviour** | **0-8hrs** | **>8hrs** |
|  |  | 1.01 (1.00-1.01) | 1.04 (1.03-1.05) |
|  | **TV viewing** | **0-3.5hrs** | **>3.5hrs** |
|  |  | 1.03 (1.01-1.04) | 1.06 (1.05-1.08) |
| **CVD mortality** | **Total Sedentary behaviour** | **0-6hrs** | **>6hrs** |
|  |  | 1.01 (0.99-1.02) | 1.04 (1.03-1.04) |
|  | **TV viewing** | **0-4hrs** | **>4hrs** |
|  |  | 1.02 (0.99-1.04) | 1.08 (1.05-1.12) |

Appendix Figure 5 - Funnel Plots with pseudo 95% confidence limits and Egger’s test for fully adjusted analyses with at least 5 contributing studies

| Appendix Table 6 – sensitivity analyses using linear PA adjusted associations, except where stated. | | | | | | | | | |  |
| --- | --- | --- | --- | --- | --- | --- | --- | --- | --- | --- |
| All-cause mortality | | | | | | | | | |  |
|  | RR | 95%CI | | Number of studies | I^2^ | | | p-value (RR=1) | |  |
| Total sedentary behaviour | | | | | | | | | |  |
| **Summary Estimate** | **1.02** | **1.01-1.03** | | **13** | **65.6** | | | **<0.001** | |  |
| ***Subgroup analysis:*** |  |  |  |  |  | | |  | |  |
| Male | 1.01 | 1.01-1.02 | | 6 | 70.9 | | | <0.001 | |  |
| Female | 1.02 | 1.01-1.03 | | 7 | 89.0 | | | <0.001 | |  |
| Non BMI adjusted* | 1.02 | 1.01-1.03 | | 9 | 74.0 | | | <0.001 | |  |
| BMI adjusted* | 1.02 | 1.01-1.03 | | 12 | 63.9 | | | <0.001 | |  |
| ***Sensitivity analysis excluding the following studies:*** | | | | | | | | | |  |
| Objectively measured sedentary time | 1.02 | 1.01-1.03 | | 11 | 69.1 | | | <0.001 | |  |
| Mean age baseline<50 | 1.02 | 1.01-1.03 | | 10 | 64.0 | | | <0.001 | |  |
| Insufficient control of baseline health | 1.02 | 1.01-1.03 | | 10 | 33.6 | | | 0.001 | |  |
| With follow-up <5yrs | 1.02 | 1.01-1.03 | | 9 | 69.2 | | | 0.001 | |  |
| Unrepresentative population | 1.02 | 1.02-1.03 | | 10 | 70.0 | | | <0.001 | |  |
| With n<10000 | 1.02 | 1.01-1.03 | | 9 | 56.2 | | | <0.001 | |  |
|  |  |  | |  |  | | |  | |  |
| TV viewing |  |  |  |  |  |  | | |  | |
| **Summary Estimate** | **1.05** | **1.04-1.05** | | **7** | **0** | | | **<0.001** | |  |
| ***Subgroup analysis:*** |  |  |  |  |  | | |  | |  |
| Non BMI adjusted* | 1.06 | 1.04-1.08 | | 9 | 76.9 | | | <0.001 | |  |
| BMI adjusted* | 1.05 | 1.02-1.08 | | 5 | 32.9 | | | 0.003 | |  |
| ***Sensitivity analysis excluding the following studies:*** | | | | | | | | | |  |
| Mean age baseline<50 | 1.05 | 1.04-1.05 | | 5 | 0 | | | <0.001 | |  |
| With follow-up <5yrs | 1.05 | 1.04-1.05 | | 6 | 0 | | | <0.001 | |  |
| Unrepresentative population | 1.04 | 1.01-1.06 | | 4 | 0 | | | 0.003 | |  |
| Studies with n<10000 | 1.05 | 1.03-1.06 | | 5 | 16.6 | | | <0.001 | |  |
|  |  |  |  |  |  | | |  | |  |
| CVD Mortality | | | | | | | | | |  |
|  | RR | 95%CI | | Number of studies | I^2^ | | | p-value RR=1 | |  |
| Total sedentary behaviour | | | | | | | | | |  |
| **Summary Estimate** | **1.02** | **1.01-1.03** | | **5** | **22.7** | | | **0.004** | |  |
| ***Subgroup analysis*** |  |  |  |  |  | | |  | |  |
| Male | 1.02 | 1.00-1.03 | | 4 | 75.6 | | | 0.017 | |  |
| Female | 1.03 | 1.01-1.04 | | 4 | 82.4 | | | 0.001 | |  |
| BMI adjusted* | 1.01 | 1.00-1.02 | | 4 | 0 | | | 0.020 | |  |
| ***Sensitivity analysis excluding the following studies:*** | | | | | | | |  | |  |
| Mean age baseline<50 | 1.01 | 1.00-1.02 | | 4 | 0 | | | <0.001 | |  |
| Insufficient control of baseline health | 1.01 | 1.00-1.02 | | 4 | 0 | | | 0.008 | |  |
| TV viewing | | | | | | | | | |  |
| **Summary Estimate** | **1.04** | **1.01-1.08** | | **6** | **88.8** | | | **0.028** | |  |
| ***Subgroup analysis*** |  |  |  |  |  | | |  | |  |
| Non-BMI adjusted | 1.05 | 1.02-1.08 | | 6 | 83.1 | | | 0.002 | |  |
| ***Sensitivity analysis excluding the following studies:*** | | | | | | | | | |  |
| Mean age baseline<50 | 1.08 | 1.06-1.10 | | 4 | 0 | | | <0.001 | |  |
| Unrepresentative population | 1.03 | 1.00-1.05 | | 4 | 16.5 | | | 0.047 | |  |
| Studies with n<10000 | 1.05 | 1.00-1.10 | | 4 | 91.2 | | | 0.051 | |  |
|  |  |  |  |  |  | |  |  |  |  |
| Cancer Mortality | | | | | | | | | |  |
|  | RR | 95%CI | | Number of studies | I^2^ | | | p-value RR=1 | |  |
| Total sedentary behaviour | | | | | | | | | |  |
| **Summary Estimate** | **1.01** | **1.00-1.02** | | **5** | **77.3** | | | **0.268** | |  |
| ***Subgroup analysis*** |  |  |  |  |  | | |  | |  |
| Male | 1.00 | 0.99-1.01 | | 4 | 0 | | | 0.856 | |  |
| Female | 1.01 | 1.00-1.02 | | 4 | 76.5 | | | 0.280 | |  |
| BMI adjusted | 1.01 | 1.01-1.02 | | 4 | 0 | | | 0.001 | |  |
| ***Sensitivity analysis excluding the following studies:*** | | | | | | | | | |  |
| Mean age baseline<50 | 1.01 | 0.99-1.02 | | 4 | 82.8 | | | 0.227 | |  |
| Insufficient control of baseline health | 1.01 | 0.99-1.02 | | 4 | 82.8 | | | 0.277 | |  |
|  |  |  | |  |  | | |  | |  |
| TV viewing | | | | | | | | | |  |
| **Summary Estimate** | **1.02** | **1.01-1.02** | | **4** | **0** | | | **<0.001** | |  |
| ***Subgroup analysis*** |  |  |  |  |  | | |  | |  |
| Non-BMI adjusted | 1.02 | 1.01-1.03 | | 5 | 0 | | | <0.001 | |  |
| ***Sensitivity analysis excluding the following studies:*** | | | | | | | | | |  |
| Type 2 diabetes | | | | | | | | | |  |
|  | RR | 95%CI | | Number of studies | I^2^ | | | p-value RR=1 | |  |
| Total sedentary behaviour | | | | | | | | | |  |
| **Summary Estimate** | **1.01** | **1.00-1.01** | | **4** | **0** | | | **<0.001** | |  |
| ***Subgroup analysis*** |  |  | |  |  | | |  | |  |
|  |  |  | |  |  | | |  | |  |
| ***Sensitivity analysis excluding the following studies:*** | | | | | | | | | |  |
|  |  |  | |  |  | | |  | |  |
| TV viewing | | | | | | | | | |  |
| **Summary Estimate** | **1.09** | **1.07-1.12** | | **6** | **31.7** | | | **<0.001** | |  |
| ***Subgroup analysis*** |  |  | |  |  | | |  | |  |
| Non BMI adjusted* | 1.09 | 1.07-1.10 | | 6 | 0 | | | <0.001 | |  |
| BMI adjusted* | 1.08 | 1.05-1.10 | | 4 | 0 | | | <0.001 | |  |
| ***Sensitivity analysis excluding the following studies:*** | | | | | | | | | |  |
| Objectively measured T2D | 1.08 | 1.07-1.10 | | 4 | 78.9 | | | <0.001 | |  |
| Follow-up <5yrs | 1.10 | 1.07-1.13 | | 4 | 48.6 | | | <0.001 | |  |
| Studies with n<10000 | 1.10 | 1.07-1.13 | | 4 | 51.4 | | | <0.001 | |  |
| *included studies might not be PA adjusted | | | | | | | | | |  |
|  | | | | | | | | | |  |

8-71)

| Appendix Table 7 - full output from the PA adjusted non-linear dose response meta-analysis for the associations of total sitting with health outcomes. RRs given for each dose for which there was a contributing data point. | | | | | | | | | | | | | | | | | | |
| --- | --- | --- | --- | --- | --- | --- | --- | --- | --- | --- | --- | --- | --- | --- | --- | --- | --- | --- |
| All-cause mortality | | | |  | CVD mortality | | | |  | Cancer mortality | | | |  | Type 2 Diabetes | | | |
| dose h/day | RR | LCI | UCI |  | dose h/day | RR | LCI | UCI |  | dose h/day | RR | LCI | UCI |  | dose h/day | RR | LCI | UCI |
| 1.5 | 1.00 | 1.00 | 1.00 |  | 0 | 1.00 | 1.00 | 1.00 |  | 1.5 | 1.00 | 1.00 | 1.00 |  | 3 | 1.00 | 1.00 | 1.00 |
| 1.86 | 1.00 | 1.00 | 1.00 |  | 0.5 | 1.00 | 0.99 | 1.01 |  | 2 | 1.00 | 0.99 | 1.01 |  | 3.5 | 1.01 | 1.00 | 1.02 |
| 2 | 1.00 | 1.00 | 1.00 |  | 1.5 | 1.00 | 0.98 | 1.03 |  | 2.5 | 1.00 | 0.99 | 1.01 |  | 4 | 1.02 | 0.99 | 1.04 |
| 2.5 | 1.00 | 1.00 | 1.01 |  | 2 | 1.01 | 0.97 | 1.04 |  | 4 | 1.00 | 0.98 | 1.03 |  | 7 | 1.06 | 0.98 | 1.15 |
| 2.88 | 1.00 | 1.00 | 1.01 |  | 2.5 | 1.01 | 0.97 | 1.05 |  | 5.5 | 1.01 | 0.97 | 1.05 |  | 8 | 1.08 | 0.99 | 1.17 |
| 3 | 1.00 | 1.00 | 1.01 |  | 4 | 1.02 | 0.95 | 1.09 |  | 6 | 1.01 | 0.96 | 1.06 |  | 9 | 1.09 | 0.99 | 1.19 |
| 4 | 1.01 | 0.99 | 1.02 |  | 5 | 1.03 | 0.95 | 1.12 |  | 7.5 | 1.02 | 0.96 | 1.08 |  | 9.5 | 1.09 | 1.00 | 1.19 |
| 4.79 | 1.01 | 0.99 | 1.03 |  | 6 | 1.05 | 0.96 | 1.15 |  | 8 | 1.02 | 0.96 | 1.08 |  | 11 | 1.10 | 1.02 | 1.18 |
| 5.5 | 1.02 | 1.00 | 1.04 |  | 7.5 | 1.09 | 0.99 | 1.20 |  | 8.5 | 1.02 | 0.96 | 1.09 |  | 12 | 1.10 | 1.03 | 1.17 |
| 6 | 1.02 | 1.00 | 1.05 |  | 8 | 1.11 | 1.01 | 1.22 |  | 9.5 | 1.03 | 0.97 | 1.10 |  | 12.5 | 1.10 | 1.04 | 1.17 |
| 6.43 | 1.03 | 1.00 | 1.06 |  | 9.5 | 1.17 | 1.06 | 1.29 |  | 10.5 | 1.04 | 0.97 | 1.12 |  | 13.5 | 1.10 | 1.03 | 1.17 |
| 6.86 | 1.04 | 1.01 | 1.06 |  | 10.5 | 1.22 | 1.10 | 1.34 |  | 11.5 | 1.05 | 0.98 | 1.14 |  |  |  |  |  |
| 7.13 | 1.04 | 1.01 | 1.07 |  | 12 | 1.29 | 1.16 | 1.44 |  | 12 | 1.06 | 0.98 | 1.15 |  |  |  |  |  |
| 7.5 | 1.05 | 1.02 | 1.08 |  | 12.5 | 1.32 | 1.18 | 1.47 |  | 12.5 | 1.07 | 0.98 | 1.16 |  |  |  |  |  |
| 8 | 1.06 | 1.03 | 1.09 |  | 13.5 | 1.38 | 1.22 | 1.55 |  | 13.5 | 1.08 | 0.98 | 1.18 |  |  |  |  |  |
| 8.5 | 1.07 | 1.04 | 1.11 |  | 16 | 1.53 | 1.32 | 1.77 |  | 16 | 1.11 | 0.98 | 1.25 |  |  |  |  |  |
| 8.86 | 1.09 | 1.05 | 1.13 |  |  |  |  |  |  |  |  |  |  |  |  |  |  |  |
| 9.5 | 1.11 | 1.06 | 1.15 |  |  |  |  |  |  |  |  |  |  |  |  |  |  |  |
| 10.26 | 1.14 | 1.09 | 1.19 |  |  |  |  |  |  |  |  |  |  |  |  |  |  |  |
| 10.5 | 1.15 | 1.09 | 1.20 |  |  |  |  |  |  |  |  |  |  |  |  |  |  |  |
| 11.5 | 1.19 | 1.13 | 1.26 |  |  |  |  |  |  |  |  |  |  |  |  |  |  |  |
| 12 | 1.22 | 1.15 | 1.29 |  |  |  |  |  |  |  |  |  |  |  |  |  |  |  |
| 12.5 | 1.24 | 1.16 | 1.33 |  |  |  |  |  |  |  |  |  |  |  |  |  |  |  |
| 13.47 | 1.30 | 1.20 | 1.40 |  |  |  |  |  |  |  |  |  |  |  |  |  |  |  |
| 13.5 | 1.30 | 1.20 | 1.40 |  |  |  |  |  |  |  |  |  |  |  |  |  |  |  |
| 13.74 | 1.31 | 1.21 | 1.42 |  |  |  |  |  |  |  |  |  |  |  |  |  |  |  |
| 14.66 | 1.36 | 1.25 | 1.49 |  |  |  |  |  |  |  |  |  |  |  |  |  |  |  |
| 15.84 | 1.43 | 1.29 | 1.59 |  |  |  |  |  |  |  |  |  |  |  |  |  |  |  |
| 16 | 1.44 | 1.30 | 1.60 |  |  |  |  |  |  |  |  |  |  |  |  |  |  |  |

| Appendix Table 8 - full output from the PA adjusted non-linear dose response meta-analysis for the associations of TV viewing with health outcomes. RRs given for each dose for which there was a contributing data point. | | | | | | | | | | | | | | | | | | |
| --- | --- | --- | --- | --- | --- | --- | --- | --- | --- | --- | --- | --- | --- | --- | --- | --- | --- | --- |
| All-cause mortality | | | |  | CVD mortality | | | |  | Cancer mortality | | | |  | Type 2 Diabetes | | | |
| dose h/day | RR | LCI | UCI |  | dose h/day | RR | LCI | UCI |  | dose h/day | RR | LCI | UCI |  | dose h/day | RR | LCI | UCI |
| 0.5 | 1.00 | 1.00 | 1.00 |  | 0.5 | 1.00 | 1.00 | 1.00 |  | 0.5 | 1.00 | 1.00 | 1.00 |  | 0.07 | 1.00 | 1.00 | 1.00 |
| 0.57 | 1.00 | 1.00 | 1.00 |  | 1 | 1.00 | 0.99 | 1.01 |  | 1 | 1.01 | 1.00 | 1.02 |  | 0.5 | 1.06 | 1.04 | 1.08 |
| 1 | 1.01 | 1.00 | 1.02 |  | 1.25 | 1.00 | 0.98 | 1.02 |  | 1.25 | 1.01 | 0.99 | 1.03 |  | 0.57 | 1.07 | 1.04 | 1.10 |
| 1.25 | 1.01 | 1.00 | 1.03 |  | 2 | 1.00 | 0.96 | 1.05 |  | 2 | 1.03 | 0.99 | 1.06 |  | 0.79 | 1.10 | 1.06 | 1.14 |
| 1.5 | 1.02 | 1.00 | 1.04 |  | 3 | 1.02 | 0.96 | 1.09 |  | 3 | 1.05 | 0.99 | 1.10 |  | 1 | 1.13 | 1.08 | 1.19 |
| 1.64 | 1.02 | 1.00 | 1.04 |  | 3.05 | 1.02 | 0.96 | 1.10 |  | 3.05 | 1.05 | 0.99 | 1.10 |  | 1.5 | 1.21 | 1.13 | 1.30 |
| 2 | 1.03 | 1.01 | 1.06 |  | 4 | 1.06 | 0.97 | 1.16 |  | 4 | 1.07 | 1.01 | 1.13 |  | 1.79 | 1.25 | 1.16 | 1.36 |
| 2.21 | 1.04 | 1.01 | 1.07 |  | 4.15 | 1.07 | 0.98 | 1.18 |  | 4.15 | 1.07 | 1.01 | 1.14 |  | 2 | 1.29 | 1.18 | 1.41 |
| 2.36 | 1.04 | 1.01 | 1.08 |  | 5 | 1.13 | 1.01 | 1.26 |  | 5 | 1.09 | 1.02 | 1.17 |  | 2.14 | 1.31 | 1.19 | 1.44 |
| 2.5 | 1.05 | 1.02 | 1.08 |  | 6 | 1.21 | 1.05 | 1.39 |  | 6 | 1.12 | 1.04 | 1.20 |  | 2.5 | 1.36 | 1.23 | 1.51 |
| 3 | 1.07 | 1.03 | 1.11 |  | 6.5 | 1.26 | 1.08 | 1.47 |  | 7 | 1.15 | 1.06 | 1.25 |  | 2.7 | 1.39 | 1.25 | 1.55 |
| 3.05 | 1.07 | 1.03 | 1.12 |  | 7 | 1.31 | 1.11 | 1.56 |  | 8 | 1.18 | 1.07 | 1.29 |  | 3 | 1.43 | 1.28 | 1.61 |
| 3.5 | 1.09 | 1.04 | 1.14 |  | 8 | 1.43 | 1.16 | 1.76 |  |  |  |  |  |  | 3.5 | 1.50 | 1.33 | 1.69 |
| 4 | 1.12 | 1.06 | 1.18 |  |  |  |  |  |  |  |  |  |  |  | 4 | 1.56 | 1.38 | 1.77 |
| 4.15 | 1.13 | 1.07 | 1.19 |  |  |  |  |  |  |  |  |  |  |  | 4.29 | 1.59 | 1.41 | 1.80 |
| 5 | 1.18 | 1.11 | 1.26 |  |  |  |  |  |  |  |  |  |  |  | 4.5 | 1.62 | 1.43 | 1.83 |
| 6 | 1.26 | 1.16 | 1.37 |  |  |  |  |  |  |  |  |  |  |  | 4.8 | 1.65 | 1.45 | 1.86 |
| 7 | 1.35 | 1.22 | 1.48 |  |  |  |  |  |  |  |  |  |  |  | 5 | 1.67 | 1.47 | 1.89 |
| 8 | 1.44 | 1.28 | 1.62 |  |  |  |  |  |  |  |  |  |  |  | 6 | 1.75 | 1.53 | 2.01 |
|  |  |  |  |  |  |  |  |  |  |  |  |  |  |  | 7 | 1.83 | 1.56 | 2.16 |
|  |  |  |  |  |  |  |  |  |  |  |  |  |  |  | 7.14 | 1.85 | 1.56 | 2.19 |
|  |  |  |  |  |  |  |  |  |  |  |  |  |  |  | 7.8 | 1.90 | 1.56 | 2.31 |
